# Assessing Computational Methods for Transcription Factor Target Gene Identification Based on ChIP-seq Data

**DOI:** 10.1371/journal.pcbi.1003342

**Published:** 2013-11-21

**Authors:** Weronika Sikora-Wohlfeld, Marit Ackermann, Eleni G. Christodoulou, Kalaimathy Singaravelu, Andreas Beyer

**Affiliations:** 1 Biotechnology Center, TU Dresden, Dresden, Germany; 2 Center for Regenerative Therapies Dresden, Dresden, Germany; 3 University of Cologne, Cologne, Germany; National Institutes of Health (NIH), United States of America

## Abstract

Chromatin immunoprecipitation coupled with deep sequencing (ChIP-seq) has great potential for elucidating transcriptional networks, by measuring genome-wide binding of transcription factors (TFs) at high resolution. Despite the precision of these experiments, identification of genes directly regulated by a TF (target genes) is not trivial. Numerous target gene scoring methods have been used in the past. However, their suitability for the task and their performance remain unclear, because a thorough comparative assessment of these methods is still lacking. Here we present a systematic evaluation of computational methods for defining TF targets based on ChIP-seq data. We validated predictions based on 68 ChIP-seq studies using a wide range of genomic expression data and functional information. We demonstrate that peak-to-gene assignment is the most crucial step for correct target gene prediction and propose a parameter-free method performing most consistently across the evaluation tests.

## Author Summary

Transcription factors (TFs) are the main regulators of gene transcription. Thus, knowing the genes that are targeted by a specific TF is of utmost importance for understanding developmental processes, cellular stress response, or disease etiology. Chromatin immunoprecipitation coupled with deep sequencing (ChIP-seq) allows for measuring the genome-wide binding of TFs. Several computational methods have been used for inferring the genes that are targeted by TFs using this binding information, but a thorough evaluation of their performance has not been performed so far. Here we present an assessment of a range of TF-target-calling methods using 68 ChIP-seq datasets. It turns out that the first step of the scoring, the assignment of binding events to genes, is the most important for correctly calling target genes. Our evaluation revealed important performance differences between the target-calling methods, with some simplistic methods exhibiting a particularly poor performance compared to more elaborate scorings. One of the methods is particularly attractive, because it does not require the *a priori* definition of any parameter — all parameters are ‘learned’ from the data. This and other methods tested were implemented in a freely available software package for future testing and application to other ChIP-seq datasets.

## Introduction

Chromatin immunoprecipitation coupled with high-throughput DNA sequencing (ChIP-seq) is a powerful technique for the genome-wide profiling of protein-DNA interactions, histone modifications, and nucleosome positions [1]. One of the most prominent applications of this technology is the genome-wide discovery of transcription factor (TF) binding sites. The procedure of identifying TF binding sites from ChIP-seq experiments is known as ‘peak calling’ and the identified genomic regions that potentially contain true TF binding sites are called ‘peaks’ [2]. Whereas a lot of effort has been put into development of peak-calling methods [3]–[5], prediction of TF targets from ChIP-seq data is still an unresolved problem [6]–[8]. Yet, this last step is critical for the functional interpretation of the many TF ChIP-seq studies that are currently being conducted.

Published ChIP-seq studies often use ad-hoc approaches for calling target genes, such as defining as targets all genes with a peak within a certain distance from the transcription start site (TSS) [9]–[11]. However, different TFs display various geometries and frequencies of binding events around a TSS for triggering transcriptional changes of the respective target gene. For example, some TFs bind at enhancer elements, which can be far from the TSS, whereas others bind close to or at proximal promoters. Such TF-specific features need to be taken into account when calling targets based on ChIP-seq data. Hence, more sophisticated approaches have been developed, which summarize all peaks around a TSS and weight each peak based on its genomic distance and/or strength [7], [8], [12], [13]. These more recent methods compute a quantitative target score reflecting the confidence that a given gene is a target based on the pattern of binding events around the TSS. Generally, it is assumed that (1) genes with many peaks in proximity to their TSS are more likely to be targets and (2) peak proximity to the TSS increases the probability of the gene being a target. These assumptions are implicitly made in virtually all published scoring schemes although they may not always hold (especially in case of enhancer elements). Published methods differ with respect to how they assign peaks to genes, how they score the peaks, and how they summarize scores of peaks in the proximity of a given TSS. The suitability of these assumptions for the task and the performance of the various scoring schemes remain unclear, because a thorough comparative assessment of these methods is still lacking. For the same reason, the key aspects that need to be tuned to improve the accuracy of target predictions remain elusive.

In this study, we systematically compared different TF target prediction approaches. We focused on the genome-wide prediction of targets using ChIP-seq data alone, because it is often the case that only this data is available. ChIP-seq-based target scores can be later integrated with expression data or any other information that could enhance target identification, if such data is available [14]. Many published methods differ with respect to implementation details such as window sizes used or how distributions of peaks around TSSs are computed. Instead of trying to compare all published implementations we decided to evaluate different strategies, like using windows versus ‘window-free’ approaches. This assessment is based on a representative selection of published methods and additional variants that allowed us to systematically evaluate the importance of individual steps. In order to facilitate such systematic analysis, we structured the target scoring into three major steps and discuss in this paper different ways of choosing the peaks to be scored, using different scoring criteria and different ways of integrating (combining) peak scores. We developed assessment procedures for evaluating TF-target prediction methods based on the consistency of the target predictions, expression data, and functional homogeneity of the predicted targets. We tested the target scorings using 68 ChIP-seq experiments conducted in mouse hematopoietic and embryonic stem (ES) cells [12], [15] comprising 42 different transcription factors (TFs), and we evaluated them across 23 expression datasets. We have implemented all the target scoring methods that we tested in a unified R-package, which can be downloaded from www.cellularnetworks.org. This package greatly facilitates the parallel testing of the various methods on any given ChIP-seq dataset.

## Results

### TF target prediction methods

**Figure 1:**
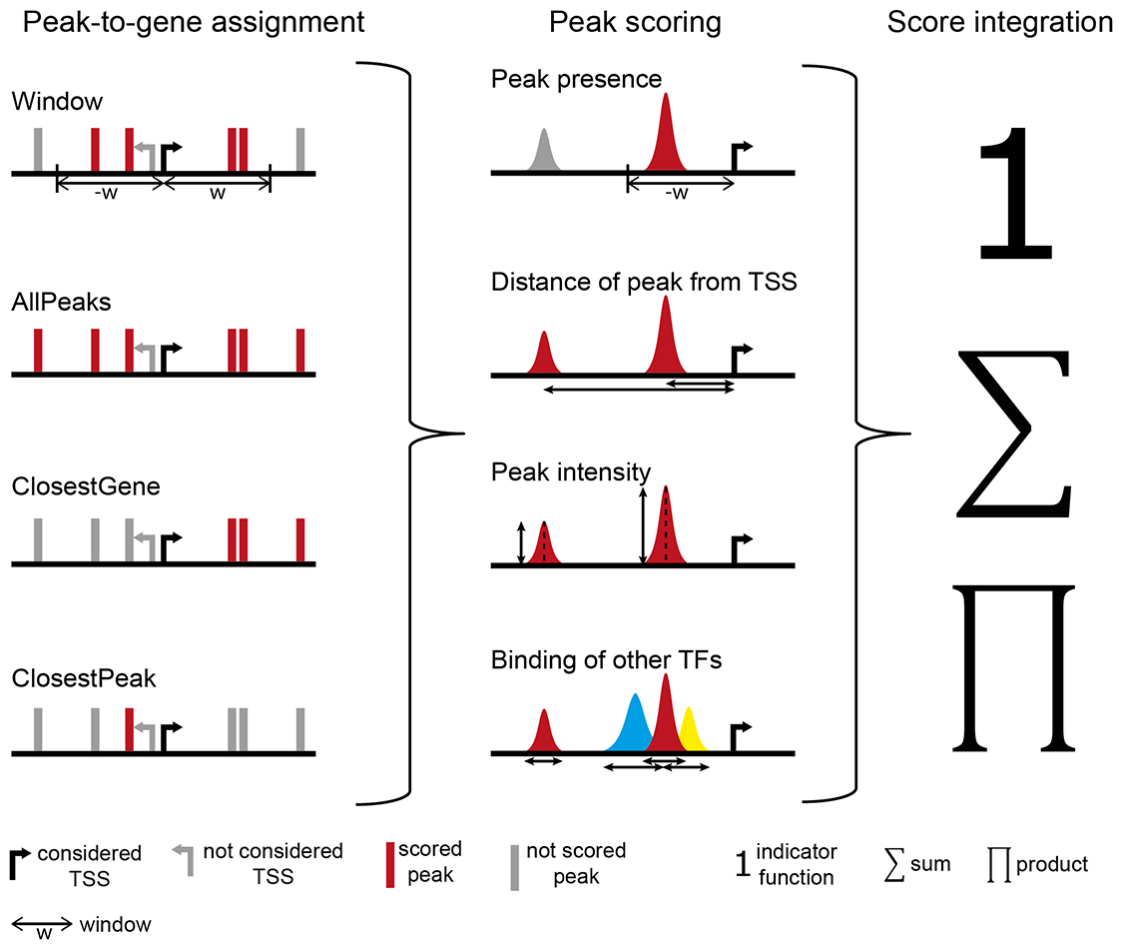
Overview of the scoring procedure. Target gene scoring consists of three steps: (1) peak-to-gene assignment, (2) peak scoring and (3) integration of individual peak scores. Black arrow indicates transcription start site (TSS) of the gene that is to be scored. Grey arrow indicates a TSS of another gene (currently not scored). Red color indicates peaks that are assigned to the evaluated (black) gene; grey peaks are not assigned to this TSS by the given peak-to-gene assignment method. Blue and yellow peaks are peaks of other TFs that might be used to score the functionality of binding sites. See Table 1 for details about the alternative scoring options.

We have structured the TF-target scoring based on ChIP-seq data into three main steps that are conducted on a gene-by-gene basis (Figure 1): (1) peak-to-gene assignment (deciding on which peaks the score of a gene is based on); (2) peak scoring (giving quantitative weights to peaks, e.g. based on their distance from the TSS); and (3) summarizing peak scores of all peaks assigned to a given gene. Target prediction methods can be classified with respect to how they implement each of these three steps. We compared six different approaches, in most cases inspired by published algorithms or ideas (Table 1). These methods were chosen to represent different types of existing approaches and to facilitate the assessment of different parameters.

**Table 1:** Characterization of TF-target prediction methods.

Method	Peak-to-gene assignment	Peak scoring and score integration	Number of peaks	Peaks positions	Peaks intensities	TF binding characteristics	Parameter free	Published algorithm
Binary	Window [−5 kb, +5 kb]	1 if at least one peak within the window, 0 otherwise	no	no	no	no	no	
Linear	Window [−50 kb, +50 kb]	Weighted sum of peaks within the window, with weights linearly decreasing with the distance from the TSS	yes	yes	no	no	no	
Ouyang (TFAS)	AllPeaks up to 1 Mb from the TSS	Sum of peak intensities weighted by the distance from the TSS, with weights exponentially decreasing with the distance from the TSS	yes	yes	yes	no	no	[13]
Cheng (TIP)	Window [−10 kb, +10 kb]	Scoring derived from observed binding profile across all TSSs	yes	yes	yes	yes	no	[8]
Chen	ClosestPeak	Proportion of non-random binding at the observed distance from the TSS	no	yes	no	yes	yes	[12]
ClosestGene	ClosestGene up to 1 Mb from the TSS	Sum of the scores based on the all-peaks-to-all-genes distance distribution	yes	yes	no	yes	yes	

#### Peak-to-gene assignment

We distinguish window-based and window-free peak-to-gene assignment methods. The simplest approach is to define a fixed window around each TSS and assign all peaks within this window to the corresponding gene (‘Window’, Figure 1) [8]–[11]. The main drawback of this method is the necessity of choosing the size of the window, which is often an arbitrary decision that does not account for binding specificities of individual TFs. Here we compare three alternatives that do not require the definition of a window: (1) assigning all peaks of the chromosome to a given gene (‘AllPeaks’, Figure 1) [12], (2) assigning each gene to its closest peak (‘ClosestPeak’, Figure 1) [13], (3) assigning each peak to its closest gene (‘ClosestGene’, Figure 1) [16], [17]. (Note the difference of the latter two.)

#### Peak scoring

In the simplest approach, which we refer to as *Binary* (Table 1), each gene with at least one peak within the window will get a score of 1, and all other genes will get a score of 0. Apart from the necessity of arbitrarily choosing the window size, this basic method has several caveats: it neither accounts for the number of peaks in the proximity of the TSS, nor their relative distances to the TSS or their intensities. More sophisticated methods use the distance of a peak from the TSS as a scoring criterion, which is based on the assumption that binding sites are more likely to influence the expression of proximal genes. A simple realization of this notion is to assign linearly decreasing weights to peaks, i.e. being ‘1’ at the TSS and ‘0’ at the far end of the window (*Linear*, Table 1), which is inspired by the study by MacIsaac *et al.* [7], who found that the influence of binding sites on expression falls off almost linearly with distance from the TSS within a 10 kb range. In addition to the distance of the peak from the TSS, Ouyang *et al.* [13] took into account peak intensity. They proposed a method (*Ouyang*, Table 1), scoring each peak based on its intensity and distance from the TSS. *Ouyang* uses AllPeaks assignment, i.e. assigns all peaks on a chromosome to a given gene, but using weights that are exponentially decaying with distance from the gene. Thus, peaks far from the TSS have negligible influence on the score. Cheng *et al.* [8] proposed an approach that implicitly accounts for peak intensity and distance from the TSS (*Cheng*, Table 1). Contrary to previously described methods, *Cheng* uses an observed binding profile within a window surrounding the TSS, taking into account individual TF binding characteristics. Another related approach also using an observed binding profile to score peaks scores each gene based on the distance to its nearest peak (*Chen*, Table 1) [12]. We compared these previously presented approaches to *ClosestGene* (Table 1), which is a novel combination of ClosestGene assignment and TF-specific distance-based peak scoring. This method summarizes the distances of peaks around all TSSs and uses the shape of the resulting distribution to assign distance-dependent scores to peaks. The underlying assumption is that the peak-to-gene distance distribution will be narrow for factors requiring binding close to the TSS, whereas it will be broader for factors that may bind further away and still affect the expression of their targets. *ClosestGene* uses the peak-to-gene distance distribution to compute the probability that a peak is found at the given distance by chance. Our implementation of this approach computes the distance distributions separately for peaks being upstream (5′) and downstream (3′) of the TSSs. Thereby it implicitly accounts for a potential bias for preferential upstream (or downstream) binding.

#### Score integration

The last step consists of integrating the scores of all peaks that have been assigned to a given gene. The *Binary* and *Chen* methods do not require any integration; *Binary* does not depend on the number of peaks and *Chen* assigns only one peak to each gene. All other methods assume an additive influence of multiple binding events on the gene expression. Therefore, the total TF-gene association score is computed as the sum of the individual scores of the peaks assigned to that gene.

### Evaluation of TF-target prediction methods

We have evaluated six target assignment methods that are representative for the different classes of methods being used in the recent literature (Table 1). For the *Binary* and *Linear* methods we tested several window sizes and chose the optimal one for the comparison with other methods (Figures S1 and S2). TF-target calling was performed using two sets of ChIP-seq experiments: The first is the HemoChIP compendium consisting of 53 ChIP-seq studies performed in mouse hematopoietic cells [15], covering 30 hematopoiesis related transcription factors or transcriptional regulators. The second set consists of ChIP-seq studies performed in mouse embryonic stem (ES) cells [12], covering 15 ES cell-related transcription factors and transcriptional regulators, which we will refer to as ESChIP. This large compendium of ChIP-seq studies covers a broad range of diverse types of transcriptional regulators and heterogeneous cell types (Table S1). For the sake of simplicity, we refer to all factors commonly as ‘transcription factors’ (TFs) although not all of them are strictly classified as specific transcription factors. Whenever possible we have evaluated the target scoring methods independently for the two datasets (HemoChIP and ESChIP) in order to assess the robustness and generality of our findings.

An obvious way to compare the performance of target scoring methods would be to use known target genes as benchmarks. However, since relatively few target genes have been validated in small scale studies, we have chosen to conduct a range of unbiased tests employing different genome-wide expression datasets and using functional consistency of the predicted targets as a criterion.

#### Perturbation expression data

First, we used perturbation expression profiling data, i.e. measurements of differential expression after inhibiting a specific TF either through RNA interference (RNAi) or genetic modifications. Functional targets of a TF are expected to respond stronger to the perturbation of a TF than non-targets. Even though perturbation experiments have indirect effects on genes that are not direct targets of the TF, a bigger overlap with perturbation expression data is indicative of better target scoring [14], [18]. We collected genome-wide perturbation expression data for 13 factors in HemoChIP and 8 factors in ESChIP (Table S1). In order to evaluate the target scorings, we compared the rankings of genes according to their target scores and according to the absolute expression fold change in response to the TF perturbation (assuming that a TF might serve as an activator or repressor for different sets of genes). Intuitively, one could compare the consistency of the whole rankings across all genes, for example calculating the Spearman rank correlation coefficient or the sum of rank products [19]. We demonstrated, however, that including all genes in the analysis leads to a subtle, but very important artifact. A large fraction of genes might not respond in any of the perturbation experiments that we used, either because they are housekeeping genes or because they were not expressed in the cell types or conditions tested. These genes would always rank low and thus any arbitrary expression dataset compared with any ChIP-seq experiment would result in better than random correlation (Figure S3). Thus, we decided to take a simple, yet, robust approach and compute the overlap (number of shared genes) between the top 500 genes based on the target scorings and the top 500 genes based on the expression profiling. Results for other sets of top scoring genes are described in Text S1. In order to correct for TF specific biases we normalized the observed overlaps for the average performance of all methods (see Materials and Methods).

**Figure 2:**
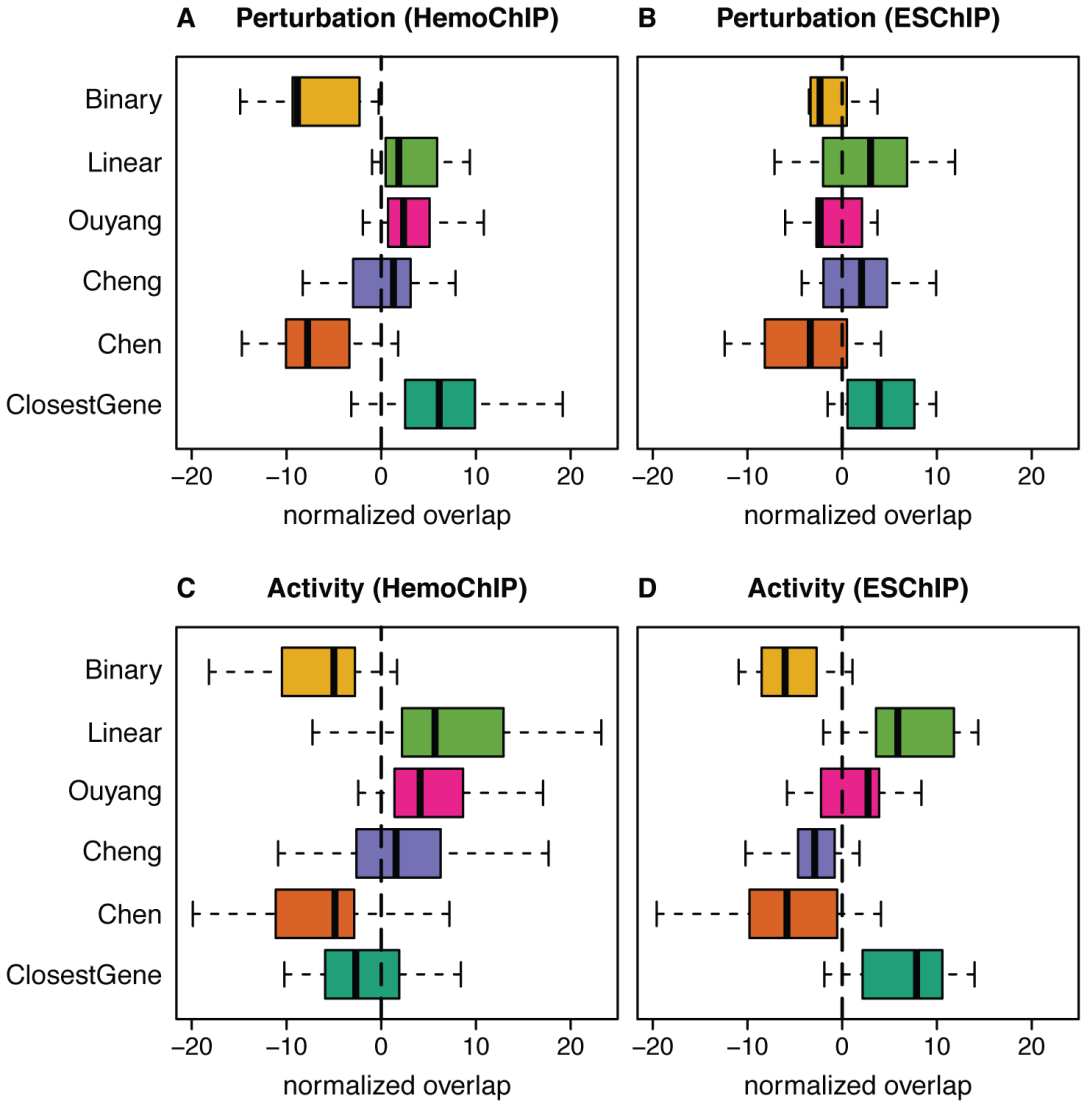
Evaluation of target scoring methods using genomic expression data. Overlap of the top 500 targets with the top 500 genes differentially expressed in (**A**) HemoChIP and (**B**) ESChIP TF perturbation experiments. Overlap of the top 500 targets with the top 500 genes differentially expressed (**C**) between erythroid and myeloid cells or (**D**) between undifferentiated (ES) and differentiated (MEF) cells. Distributions of those normalized values are shown.

The first important observation was that all methods resulted in target scorings that are consistent with the perturbation expression data. We performed permutation tests that showed that the overlap is significantly reduced when randomly picking the same number (i.e. 500) of genes (Figure S4). This gives credibility to the target scoring methods and confirms that perturbation expression data can be used for validating target predictions, even if sometimes different cell lines or cell types were used. However, we also observed important differences between the scoring methods (Figure 2 A, B). *Binary* and *Chen* performed worst suggesting that considering peak distances from the TSS (neglected by *Binary*) as well as proper peak-to-gene assignment are crucial aspects of target scoring. In addition, the *Chen* method suffers from the fact that the scoring is based on one peak only and thus is very sensitive to experimental variation in ChIP-seq data, such as miscalled peaks. The best performing method in this test was *ClosestGene* (Figure 2 A, B). Similar conclusions could be drawn when comparing other sets of target genes (Figure S5).

#### Differential TF activity data

Perturbation expression experiments yield relatively specific, functional data on genes whose expression is directly or indirectly affected by the TF. However, they mostly do not reflect physiological conditions. We therefore performed a complementary analysis using expression data of different cell types, for which we expected (based on the function of the TF) that a given TF would change its activity. As an effect true targets of the TF should exhibit differential expression between these two conditions. This latter analysis rests on more physiological conditions than engineered TF activity changes, but it has the drawback that several TFs may change their activity between the two cell states. Thus, observed expression differences cannot exclusively be attributed to the TF of interest. This analysis was performed similarly as for perturbation expression data (Figure 2 C, D). The results are mostly in agreement with the previous analysis, except that *ClosestGene* performs worse than *Linear*, *Ouyang* and *Cheng* on the HemoChIP data (Figure 2 C, D). Similar conclusions could be drawn when comparing other sets of target genes (Figure S6).

#### Functional homogeneity of targets

**Figure 3:**
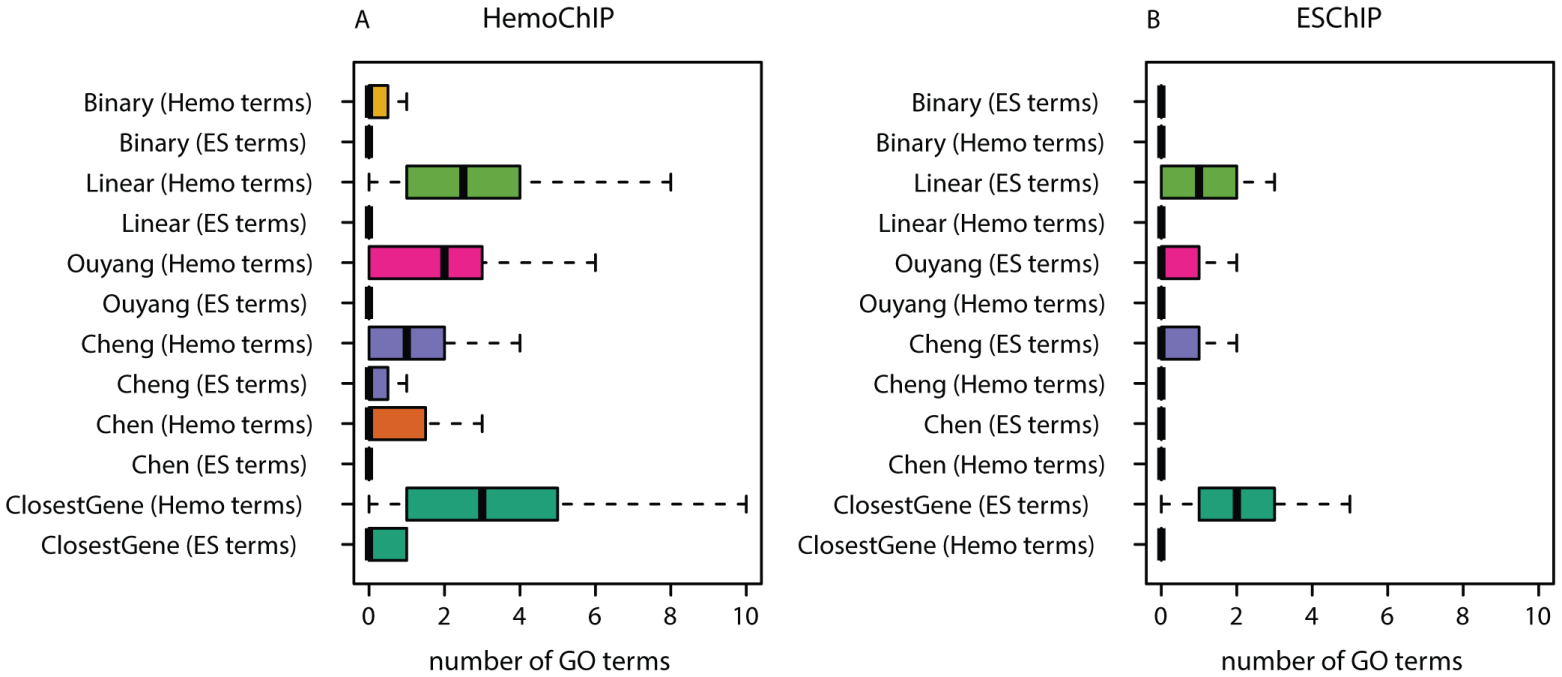
Functional homogeneity of targets. Number of significantly enriched GO terms specific for a given cellular system and specific for the opposite cellular system for HemoChIP (**A**) and ESChIP (**B**). The specific terms are hematopoiesis or embryonic development related GO terms for HemoChIP and ESChIP, respectively.

Genes being targeted by the same TF should be functionally related and a significant fraction of the target genes should be functionally related to the TF [14], [20]. In particular, we expected that targets of HemoChIP-TFs should be relevant for hematopoiesis and targets of ESChIP-TFs should be annotated for processes related to ES cell biology. Thus, methods generating target lists that are functionally more homogeneous and whose functionality is of higher relevance to the given cell type should be preferable. To quantify this notion we performed GO enrichment analyses [21], [22] on the different target scorings using the top 500 targets and compared the number of significantly enriched (p–value<0.001, Fisher's exact test) cell-type specific GO terms of the ‘biological process’ category (Figure 3). For HemoChIP we counted the hematopoiesis related GO terms, and for ESChIP the GO terms related to embryonic development and stem cell maintenance (see Materials and Methods). *ClosestGene* had the largest number of relevant GO terms in both cases (HemoChIP and ESChIP). Similar conclusions could be drawn when comparing other sets of target genes (Figure S7) or other p-value thresholds (Figure S8).

#### Consistency and specificity of target predictions

**Figure 4:**
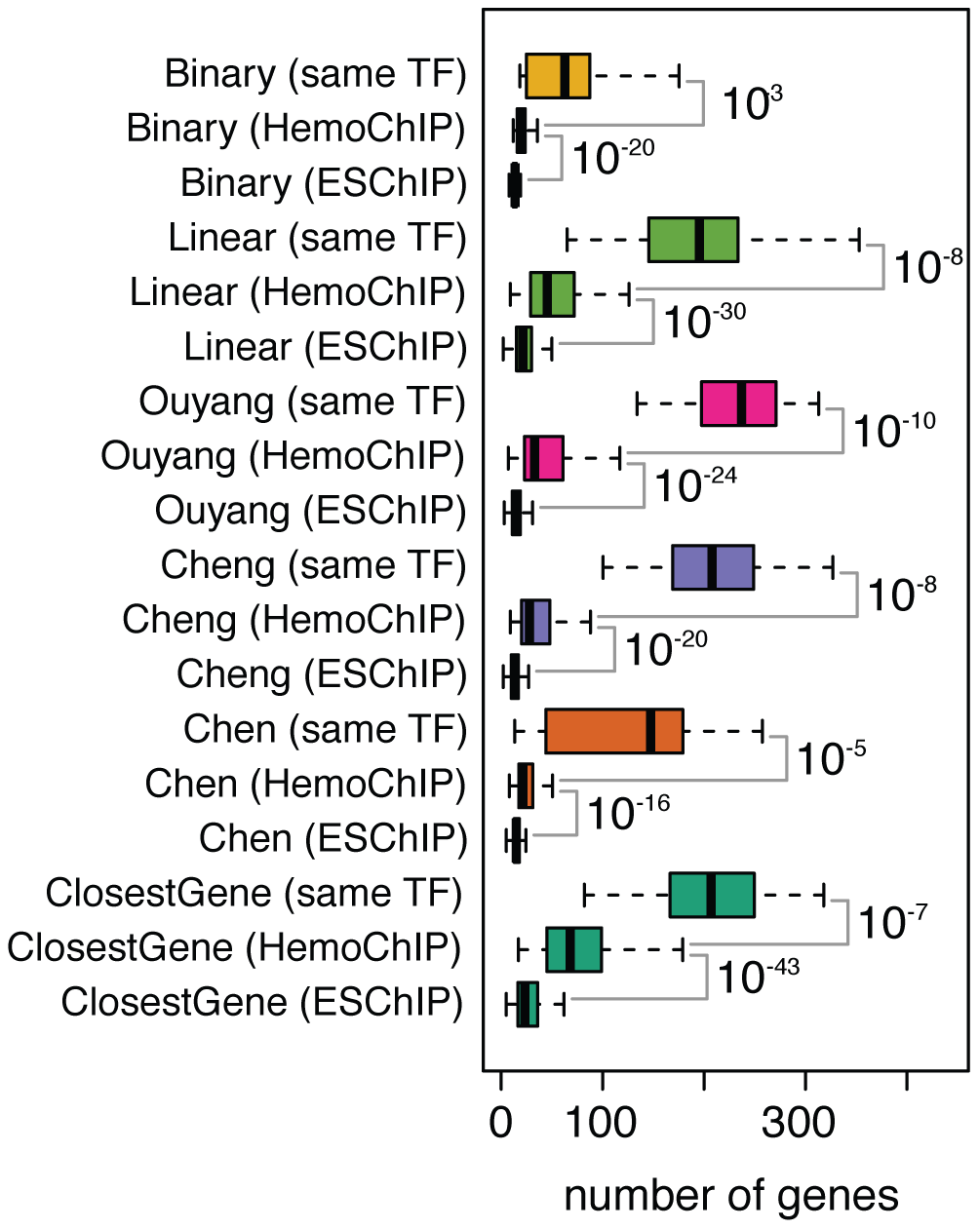
Consistency of target gene predictions. Independent ChIP-seq experiments are available for some of the factors measured in the hematopoietic system. Consistency of target predictions is quantified as the overlap between the top 500 target genes. Results are summarized based on intersecting targets from pairs of ChIP-seq experiments measuring the same transcription factor (‘same TF’), using ChIP-seq experiments from different factors, but the same system (hematopoietic cells, ‘HemoChIP’) and using ChIP-seq experiments from a different system (ES cells, ‘ESChIP’). Numbers on the right are rounded p-values, measuring the significance of the difference between the overlaps (t-test). P-values for the comparison ‘same TF’ versus ‘HemoChIP’ are generally less significant than ‘HemoChIP’ versus ‘ESChIP’, because the number of comparisons (observations) is smaller.

In order to investigate the robustness of the TF-target prediction methods, we checked the consistency of the target predictions across different ChIP-seq experiments for the same TF, available for several TFs in the HemoChIP compendium. Although these datasets are not replicates, but independent experiments, performed in different cell types or different conditions, we reasoned that targets predicted for the same TF in different studies should be more similar than targets predicted for different TFs. We thus used the consistency of targets predicted based on different ChIP-seq experiments for the same TF compared to the overlap of predictions for different TFs as a criterion to quantify the quality of the target scorings (Figure 4). In order to also assess the specificity of the predictions we distinguished the overlap between ChIP-seq studies measuring (1) the same TF, (2) measuring different TFs but also in hematopoietic cell types, and (3) measuring different TFs in a different cellular system (i.e. ESChIP, Figure 4). The overlap between ChIP-seq experiments performed on the same TF usually falls in the range of 150 to 250 out of 500 genes, which is statistically highly significant (expected number of overlapping genes if the ranking was random is on average 9.72). Perfect overlap is not expected, because the compared datasets are not replicates, but independent experiments, differing both in biological (different cell types or conditions) and technical (different labs, different antibodies, etc.) aspects. Importantly, the overlap between the ChIP-seq experiments of the same TF is significantly higher than the overlap between different factors from the HemoChIP compendium, which in turn is higher than the overlap with ChIP-seq experiments from ESChIP. Since the HemoChIP TFs are likely to share more targets between each other than with ESChIP TFs, higher overlap with HemoChIP TFs is expected. Although all TF-target prediction methods tested are robust and specific, meaning they can repeatedly identify targets of a given TF across different ChIP-seq experiments for the same TF, we observed important differences between the scoring methods. *Binary* and *Chen* performed worst in these tests, while *Ouyang* and *ClosestGene* were performing best (*Ouyang* had the highest specificity when comparing TF-specific ChIP-seq experiments *versus* other HemoChIP experiments. *ClosestGene* had the highest specificity when comparing HemoChIP experiments *versus* ESChIP experiments). The relatively weak performance of *Binary* and *Chen* correlates with the fact that they both ignore the number of peaks in the proximity of a gene. Similar conclusions could be drawn when comparing other sets of target genes (Figure S9).

#### Inclusion of additional genomic data

We and many others have shown that the consideration of additional information, such as binding site conservation, expression data, or interactions among target genes, improves TF target gene identification [14], [18], [23]–[26]. In this study we have exclusively used ChIP-seq data in order to specifically investigate the utility of different types of information that can directly be extracted from high-throughput DNA binding data. Other information can be included in subsequent steps. The scorings discussed so far rely on various aspects of TF binding, such as number of peaks in the proximity of the TSS, relative distances of the peaks from the TSSs or peak intensities. However, due to different implementations, it is difficult to directly compare the importance of these various aspects on target prediction. Therefore, we extended the *ClosestGene* method, which in its basic form uses distance between peaks and TSSs as the sole weighting criterion, and conducted additional analyses using also peak intensities and presence of co-factors as scoring criteria (see Text S1). We observed that peaks closer to TSSs tend to be stronger and that they more often co-bind with other TFs than peaks binding further away (Figures S10 and S11). Surprisingly, including these additional parameters did not improve the target gene prediction (Figure S12). The scoring that we tested was based on the number of other TFs binding at a given site. However, the relationship between co-binding (which not always implies co-activity) and functionality of a binding site may be much more complex. Approaches explicitly modeling combinatorial interactions between TFs have proven to better explain expression changes than single-TF analyses [13]. Depending on the peak calling method, peak intensities are affected by numerous factors not related to the binding strength or functionality of the binding. For example, peak intensities and their significance are affected by the local background of DNA being purified, by the mapability of the specific region and by the presence or absence of co-factors in the protein-DNA complex, which may affect the efficiency of the immune purification. These factors might partially explain why in this case the inclusion of peak intensity did not improve target predictions.

### Gene density around predicted targets

**Figure 5:**
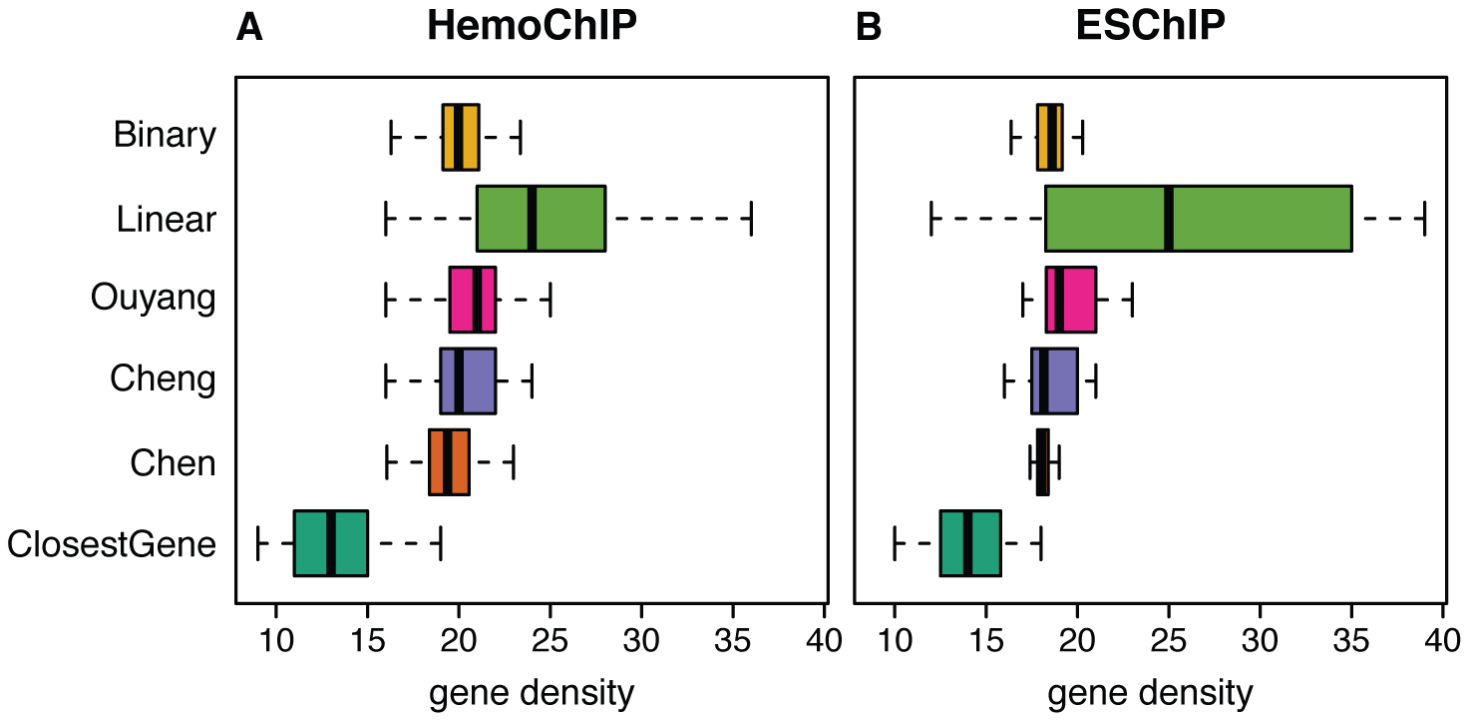
Gene density in target regions. Gene dense regions tend to contain more binding events (peaks) than gene sparse regions of the genome. The figure shows gene density (number of genes inside 1 Mb regions around the target gene's TSS) of the regions harboring the top 500 genes across the studies in the (**A**) HemoChIP and (**B**) ESChIP datasets.

Another important aspect of peak-to-gene assignment not discussed so far is its sensitivity to gene density of genomic regions. Methods using a fixed window around the TSS are prone to miscall targets in gene-rich regions. The fact that density of peaks is higher in gene-dense regions (these regions are likely to contain more targets than gene-sparse regions) increases the likelihood of finding a peak close to any gene in such region. In order to test the relevance of this issue for target gene identification we compared the gene densities around the highest scoring targets for the different scoring methods (Figure 5). The strongest gene density bias was observed for *Linear*, due to the relatively large window size (−50 kb to 50 kb) used in this method. Methods using smaller window sizes (*Binary*, *Cheng*) or relying on fewer proximal peaks (*Chen*) show a smaller bias in favor of gene-rich regions. Importantly, *Ouyang*, which assigns all peaks to each gene (but with decreasing weight) shows a stronger bias than methods considering only peaks in the proximity of the TSS. Compared to the other methods, *ClosestGene* shows a bias in favor of gene-poor regions, which can be explained by the fact that in a gene-poor region more peaks are likely to be assigned to a single gene. These gene density biases are persistent across different sets of targets (Figure S13).

## Discussion

In this study we have presented the first systematic comparison of ChIP-seq based TF-target prediction methods using a wide range of ChIP-seq studies from different biological systems. We have developed robust evaluation tests that account for several biases and potential pitfalls. Structuring the target scoring process into three phases helped systematizing the methods and it revealed the peak-to-gene assignment as the most important step. Although all methods resulted in TF-target predictions that are better than random, we observed important differences between the methods.

A common principle underlying all of these methods is the scoring of putative TF-targets by correlating the positions of TF binding sites with the positions of TSSs. Even though genomic distance is not a perfect predictor for TF-target interactions (especially in the case of enhancers and silencers) this approach yields statistically significant predictions. Individual targets would however have to be validated through functional assays. Out of six compared methods, *Binary* and *Chen* clearly performed the worst. In contrast, all other methods accounting for the number and distance of peaks around a TSS consistently performed better across all our tests. All of these methods (*Linear*, *Ouyang*, *Cheng*, *ClosestGene*) performed similarly, but we observed a small advantage of *ClosestGene* especially in the expression data tests and GO enrichment. Although the performance gain was small it was consistent when changing thresholds (see Text S1). This indicates that assigning each peak to its closest gene, a simple solution that has been used before to determine TF targets [16], yields specific and biologically relevant results. The *ClosestGene* approach presented in this study is a combination of three ideas that have not been used in this combination in the past: (1) assigning peaks to their closest gene, (2) scoring peaks based on the distribution of all peaks around the TSSs, and (3) considering (summing) all peaks assigned to a particular gene. Contrary to all other methods tested, *ClosestGene* does not require the definition of any parameters, giving it another advantage.

An obvious limitation of all methods tested herein is their inability to predict distal regulatory events. Matching long-range DNA interaction data obtained through methods such as 5C [27] or ChIA-PET [28] are currently not available in sufficient quantity to aid the interpretation of most ChIP-seq studies. We are convinced, however, that including complementary data, especially on long-range DNA interactions, and further improving the scoring by accounting for combinatorial interactions will yield even better target scorings in the future.

Another factor influencing the quality of the TF target prediction from ChIP-seq data is the quality of peak calling itself. Different peak calling algorithms result in different numbers of identified peaks and/or different peak intensities, which in turn influences the performance of target identification methods. All, but one (*Cheng*), methods assessed in this paper use the list of identified peaks as input. Since all target prediction methods were faced with the same set of peaks, the evaluation tests allow for a fair comparison of the predictions.

It should be emphasized that all of our analysis is based on mouse data. Whereas it is very plausible that the performance of the target prediction methods will be similar for other mammalian species, ChIP-seq data from non-mammalian species may have different requirements. Hence, similar assessments should be performed for other species.

## Materials and Methods

### ChIP-seq data

Fifty three ChIP-seq studies covering 30 transcription factors and transcriptional regulators were taken from HemoChIP [15]. We used the peaks as identified in the original HemoChIP publication [15]. BED files containing the peak coordinates as well as BIGWIG files were downloaded from http://hscl.cimr.cam.ac.uk/ChIP-Seq_Compendium/ChIP-Seq_Compendium2.html. For several TFs there are multiple corresponding ChIP-seq studies in the HemoChIP compendium. For those TFs we chose one representative ChIP-seq study (indicated in Table S1), on which we based the target prediction used in the majority of the analyses and evaluation tests we have conducted (we wanted to avoid biasing the results of the evaluation tests due to the overrepresentation of some TFs). We also used, however, the remaining ChIP-seq studies to evaluate the consistency of the predictions.

The ChIP-seq studies of the 15 transcription factors and transcriptional regulators performed in mouse ES cells, which we refer to as ESChIP, were obtained from [12], GSE11431. Peak calling was performed using MACS version 2.0.6 [29].

In case of both, HemoChIP and ESChIP the peak summit was used as the location parameter for distance computations. The peak intensity was calculated as the maximum pileup height extracted from BIGWIG file (HemoChIP) or pileup height at peak summit as returned by MACS (ESChIP). Peaks with intensity above 200 were excluded from the analysis.

### Expression datasets used for validation

For the perturbation analysis, 13 HemoChIP related and 8 ESChIP related expression datasets were collected (Table S1). In the selected studies the expression profile of the wild-type cells was compared to the expression profile after knock-out or knock-down of the respective TF. All measurements were conducted in mouse tissues, in most cases close to the hematopoietic or embryonic cell types. Whenever Affymetrix raw data were available, they were normalized with GCRMA, using Bioconductor [30] and Brainarray custom CDF files [31] to summarize probes to genes based on Ensembl gene IDs. Otherwise, the normalized data were downloaded, quantile normalized and 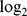 transformed when necessary, and summarized to genes using Affymetrix, Illumina or Agilent probes to Ensembl gene IDs mapping retrieved from the Ensembl64 database (www.ensembl.org) using the R package biomaRt [32]. In case of the GATA1 and PU.1 expression datasets genes with expression levels lower than the 0.3 quantile under both conditions were removed. After averaging replicates, the absolute 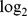 of the expression ratio between the perturbation and control conditions (fold change) was calculated for each gene.

For the activity analysis of HemoChIP TFs, the normalized expression data were obtained from [33], where the expression measurements in four different hematopoietic cell types (stem cells, progenitors, erythroid and myeloid cells) across a number of BXD mouse strains are reported. We used expression measurements from erythroid and myeloid cells, since we expected that most of the TFs in HemoChIP have different activities in these two cell types. The probes were summarized to genes based on Ensembl gene IDs and the absolute 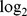 of the expression ratio between erythroid and myeloid cells (fold change) was calculated for each gene. The fold change was calculated for each strain (with measurements in both cell types) and then averaged.

For the activity analysis of ESChIP TFs, we compared expression profiles for ES cells and differentiated (MEF) cells [34], since most self-renewal and pluripotency related TFs are known to change their activity upon differentiation. The raw data were downloaded (GSE14012) and normalized with GCRMA, using Bioconductor [30] and Brainarray custom CDF files [31] to summarize probes to genes based on Ensembl gene IDs. After averaging replicates, the absolute 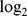 of the expression ratio between the undifferentiated and differentiated cells (fold change) was calculated for each gene.

### Gene positions

Genomic positions of the transcripts were retrieved from the Ensembl64 database (www.ensembl.org) using the R package biomaRt [32]. Only genes encoded on the autosomes and chromosome X were considered. Out of those, all genes other than the ones described as protein-, miRNA-, or lincRNA-coding were excluded. This filtering resulted in 81,881 transcripts, assigned to 25,199 genes. If multiple transcripts were assigned to one gene, the most 5′ TSS position of the transcripts was considered as the representative TSS of the gene, so that ultimately each gene was associated with one TSS only.

### TF-target prediction methods

For the *Binary* and *Linear* methods, a window of fixed size was set around each gene's representative TSS, centered at the TSS. Binding events within the window were treated equally, irrespective of whether they occurred upstream or downstream from the TSS. The window sizes were optimized based on our assessment criteria (see Figures S1 and S2).

#### *Binary*

A gene was designated as a target with a score 1 if there was at least one peak within the window, and as a non-target with a score 0 otherwise. For this approach we chose a window [−5 kb, +5 kb], i.e. spanning the region from 5 kb upstream of the TSS to 5 kb downstream of the TSS (Figure S1).

#### *Linear*

A gene's score was calculated as a weighted sum of the peaks within the window. The weights linearly decreased with distance from the TSS being 1 at the TSS and 0 at the edges of the window. The TF-gene association score of a gene 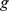 and a TF 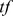 was computed as:
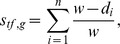
where 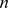 is the number of peaks within the window, 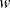 is the size of the window meaning the distance from the TSS to the edge of the window (upstream or downstream), and 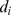 is the distance of the 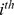 peak from the TSS. In case of *Linear* the window was set to [−50 kb, +50 kb] (Figure S2).

#### *Ouyang*

The TF association strength (TFAS) score was implemented as described in [13]. Following the original publication, the value of 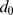 (a constant determining how strongly the peak effect decays with distance from the TSS) was set to 500 bp for E2F1 in ESChIP and 5000 bp for other TFs in ESChIP and HemoChIP.

#### *Cheng*

The target identification from profiles (TIP) method was implemented as described in [8] using the code available online http://archive.gersteinlab.org/proj/tftarget/. There are two variants of this method: (1) using a binding profile inferred directly form the wiggle file or (2) using a list of identified peaks as input. We used the first approach and following the original publication, we set the window size to [−10 kb, +10 kb] to calculate binding profile around the TSS.

#### *Chen*

The TF-gene association score was implemented as described in [12].

#### *ClosestGene*

In the first step, each peak was assigned to its closest gene. When a particular gene was scored, only the peaks assigned to that gene, i.e. peaks closer to that gene than to any other, were considered. The peak scoring was derived from the observed peak-to-gene distance distribution.

The peak-to-gene distance distribution was obtained by computing the distances between each TSS and all peaks within 1 Mb upstream and 1 Mb downstream of the TSS. Separate distributions for upstream and downstream binding were generated and the distances were pooled across all chromosomes. This distribution approximates the distribution of the distances of all peaks to all genes. The restriction of the distance to 1 Mb was applied in order to minimize artifacts for genes located close to the telomeres and due to different lengths of the chromosomes.

Subsequently, individual peaks were scored using the cumulative distance distributions by computing the fraction of peaks observed at the given distance from the TSS or closer (again separately for upstream and downstream binding). This fraction (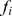) was interpreted as the probability of observing the peak at this position or closer to the TSS if the gene was not a target of the TF. The interaction score between TF 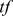 and gene 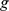 was calculated as the sum of 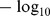 transformed scores 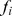 for individual peaks assigned to a given gene:
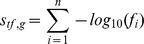



### Evaluation of the TF-target prediction methods

#### Consistency analysis

For each TF, for which multiple ChIP-seq datasets were available (HemoChIP only), the overlaps between the top 500 genes ranked according to the TF-gene score in the representative ChIP-seq dataset and the top 500 of genes ranked according to the TF-gene score in other ChIP-seq studies for the same TF were calculated. For *Binary* method, which yields discrete scores, it was not always possible to unambiguously define the top 500 genes. In the case of bindings, the top 500 genes were randomly sampled 1000 times and the average overlap was reported. We have validated that our conclusions are robust with respect to the choice of this set of top genes (Figure S9).

#### Perturbation and activity analysis

For each TF, for which perturbation expression data were available, the overlap between the top 500 genes ranked according to the TF-gene association score and the top 500 genes ranked according to the fold change between perturbation and control conditions was calculated. In case of the activity analysis respective fold changes from different conditions were used. Bindings were handled in the same way as for the consistency analysis. The overlap between the target scorings and expression data not only depends on the performance of the scoring methods but also on the compatibility between the ChIP-seq data and the expression data (e.g. depending on the cell types measured). In order to correct for this effect the average overlap achieved by all methods for a particular ChIP-seq - expression data pair was subtracted. Hence, this measures how much better (or worse) than average each method performed on a given dataset.

We have validated that our conclusions are robust with respect to the choice of this set of top genes (Figure S5 and S6).

#### GO enrichment

For each TF the GO enrichment was analyzed using the ‘biological process’ GO category. Genes were ranked according to the target score and the enrichment of top 500 genes with specific GO functions was quantified with Fisher's exact test using the ‘elim’ algorithm from the topGO R package [21]. The number of significant (p–value<0.001) hematopoiesis-specific terms (term ‘hemopoiesis’ and all its child terms) and ES cells specific terms (terms ‘embryo development’ and ‘stem cell maintenance’ and all their child terms) was reported for each ChIP-seq dataset. We have validated that our conclusions are robust with respect to the choice of the set of top genes (Figure S7) or p-value threshold (Figure S8).

## Supporting Information

Figure S1 **Performance of** ***Binary*** **for different window sizes.** Overlap of the top 500 targets with the top 500 genes differentially expressed in (**A**) HemoChIP and (**B**) ESChIP TF perturbation experiments. Overlap of the top 500 targets with the top 500 genes differentially expressed (**C**) between erythroid and myeloid cells or (**D**) between undifferentiated (ES) and differentiated (MEF) cells.
(PDF)

Figure S2 **Performance of** ***Linear*** **for different window sizes.** Overlap of the top 500 targets with the top 500 genes differentially expressed in (**A**) HemoChIP and (**B**) ESChIP TF perturbation experiments. Overlap of the top 500 targets with the top 500 genes differentially expressed (**C**) between erythroid and myeloid cells or (**D**) between undifferentiated (ES) and differentiated (MEF) cells.
(PDF)

Figure S3
**Specificity of TF-target prediction methods.** High specificity of target predictions means that there should be higher congruence with perturbation expression data from the same factor than with perturbation data from different factors. Here, we compare measuring consistency based on the overlap of the top 500 genes (**A**, **B**; considering only top ranking genes) with measuring consistency based on the sum of rank products (**C**, **D**; comparing the entire rankings). Specificity is quantified as the difference between the scorings obtained for matching data (matching ChIP-seq and expression data) versus non-matching data, expressed as the –log_10_ (p–value) of the respective t-test (horizontal axis). ‘Same category’ (**A**, **C**) refers to comparing matching and non-matching pairings from the same cellular system. ‘Other category’ (**B**, **D**) refers to comparing matching pairings with pairing HemoChIP ChIP-seq data with ES cell expression data and vice versa. Larger differences are expected in the latter case. ‘All’ using all genes for the scoring; ‘5000’ and ‘2000’ using only the top 5000 (or 2000) most variable genes. The dashed line corresponds to p-value = 0.05.(PDF)

Figure S4 **Significance of the targets recovery within top 500 genes.** Z-scores of the overlap between the top 500 targets with the top 500 genes differentially expressed in (**A**) HemoChIP and (**B**) ESChIP TF perturbation experiments, (**C**) between erythroid and myeloid cells, (**D**) between undifferentiated (ES) and differentiated (MEF) cells. The difference between this visualization and Figure 2 of the main text is that here the overlaps are not normalized for the average performance across all methods. Thus, the z-scores do not account for the fact that some ChIP-seq studies intrinsically match better with the respective expression data (e.g. because identical cell types were used) than others.
(PDF)

Figure S5 **Targets recovery within different sets of genes (perturbation).** Overlap of the top (**A,B**) 300, (**C,D**) 500 or (**E,F**) 1000 targets with the respective number of genes differentially expressed in (**A,C,E**) HemoChIP and (**B,D,F**) ESChIP TF perturbation experiments.
(PDF)

Figure S6 **Targets recovery within different sets of genes (activity).** Overlap of the top (**A,B**) 300, (**C,D**) 500 or (**E,F**) 1000 targets with the respective number of genes differentially expressed (**A,C,E**) between erythroid and myeloid cells or (**B,D,F**) between undifferentiated (ES) and differentiated (MEF) cells.
(PDF)

Figure S7 **Functional homogeneity of targets for different sets of targets.** Number of significantly enriched GO terms among top (**A,B**) 300, (**C,D**) 500 or (**E,F**) 1000 targets specific for a given cellular system and specific for the opposite cellular system for HemoChIP (**A,C,E**) and ESChIP (**B,D,F**). The specific terms are hematopoiesis or embryonic development related GO terms for HemoChIP and ESChIP, respectively.
(PDF)

Figure S8
**Functional homogeneity of the targets for different enrichment significance thresholds.** Number of significantly enriched GO terms specific for a given cellular system and specific for the opposite cellular system for HemoChIP (**A,C**) and ESChIP (**B,D**). The significantly enriched GO terms were defined as the ones with p–value<0.05 (**A,B**) or p–value<0.0001 (**C,D**) of the Fisher's exact test.(PDF)

Figure S9 **Consistency of target gene predictions for different sets of targets.** Independent ChIP-seq experiments are available for some of the factors measured in the hematopoietic system. Consistency of target predictions is quantified as the overlap between the top (**A**) 300, (**B**) 500 or (**C**) 1000 target genes. Results are summarized based on intersecting targets from pairs of ChIP-seq experiments measuring the same transcription factor (‘same TF’), using ChIP-seq experiments from different factors, but the same system (hematopoietic cells, ‘HemoChIP’) and using ChIP-seq experiments from a different system (ES cells, ‘ESChIP’). Numbers on the right are rounded p-values, measuring the significance of the difference between the overlaps (t-test). P-values for the comparison ‘same TF’ versus ‘HemoChIP’ are generally less significant than ‘HemoChIP’ versus ‘ESChIP’, because the number of comparisons (observations) is smaller.
(PDF)

Figure S10 **Characterization of binding events with different numbers of co-factors.** Density distribution of binding sites around TSSs as a function of the number of factors binding (as shown in legend). Figure shows a fragment of the density distribution plot; the density below the lines sums to 1 for distances from −1 Mb to 1 Mb from the TSS.
(PDF)

Figure S11 **Characterization of binding events with different intensities.** Density distribution of peaks around TSSs as a function of normalized peak intensity (1 = highest intensity, 0 = lowest intensity; see legend) for (**A**) HemoChIP and (**B**) ESChIP. Figure shows fragments of density distribution plots; the below the density lines sums to 1 for distances from −1 Mb to 1 Mb from the TSS.
(PDF)

Figure S12 **Combination of peak-scoring criteria.** Peaks are scored based on their distance to the TSS (Distance), intensity (Intensity) or number of co-binding factors at the same site (Co-binding) or combinations thereof (as indicated). Peak-to-gene assignment and score integration is done using the *ClosestGene* scheme. Overlap of the top 500 targets with the top 500 genes differentially expressed in (**A**) HemoChIP and (**B**) ESChIP TF perturbation experiments. Overlap of the top 500 targets with the top 500 genes differentially expressed (**C**) between erythroid and myeloid cells or (**D**) between undifferentiated (ES) and differentiated (MEF) cells.
(PDF)

Figure S13 **Gene density in target regions for different sets of targets.** Gene density (number of genes inside 1 Mb regions around the target gene's TSS) of the regions harboring the top 300 (**A, B**), 500 (**C, D**) or 1000 (**E, F**) genes across the studies in the HemoChIP (**A, C, E**) and ESChIP datasets (**B, D, F**).
(PDF)

Figure S14 **Characterization of transcriptional regulators.** Fraction of peaks binding at promoters, or co-occurring with P300 or CTCF binding. Overlap encompasses peaks falling in more than one of those categories. Regulators are grouped depending on the cellular system in which the corresponding ChIP-seq study has been conducted (hematopoietic cells or embryonic stem cells, respectively).
(PDF)

Figure S15 **Performance of** ***ClosestGene*** **for different scorings.** Z-scores of the overlap between the top 500 targets with the top 500 genes differentially expressed in (**A**) HemoChIP and (**B**) ESChIP TF perturbation experiments, (**C**) between erythroid and myeloid cells, (**D**) between undifferentiated (ES) and differentiated (MEF) cells. *ClosestGene* corresponds to *ClosestGene* using TF-specific peak-to-gene distance distribution. *ClosestGene (symmetric)* corresponds to the variant where a distribution symmetric around a TSS (obtained by pooling all peak-to-gene distances without distinguishing between upstream and downstream peaks). *ClosestGene (unspecific)* corresponds to the variant where a distribution specific for another TF is used for peak scoring. *Linear 1Mb* corresponds to the variant where peaks assigned to the TSS are scored using linearly decreasing weights.
(PDF)

Figure S16 **Peak-to-gene distance distribution.** Peak-to-gene distance distribution for (**A**) OCT4 and (**B**) P300 used for peak scoring.
(PDF)

Figure S17 **Performance of the OCT4 target prediction using different distributions.** Z-score representing the significance of the overlap between top 500 targets and top 500 genes differentially expressed after *Oct4* knock-down (‘perturbation’) or between ES and undifferentiated (MEF) cells (‘activity’) when scoring OCT4 peaks using OCT4 (‘OCT4’) or P300 (‘P300’) distributions.
(PDF)

Figure S18 **Overlap between targets and differentially expressed genes.** Median overlap between top 300, 500 and 1000 targets with the respective number of genes differentially expressed in (**A**) HemoChIP and (**B**) ESChIP TF perturbation experiments. Median overlap between top 300, 500 and 1000 targets with the respective number of genes differentially expressed (**C**) between erythroid and myeloid cells or (**D**) between undifferentiated (ES) and differentiated (MEF) cells.
(PDF)

Table S1 **Transcription factors and data included in the study.**
(PDF)

Text S1 **Supporting text.** (1) TF characterization based on ChIP-seq studies. (2) Importance of using TF-specific peak-to-gene distance distributions. (3) Incorporation of peak height or binding of co-factors does not improve target prediction. (4) Significance and robustness of evaluation methods. (5) Ranking is biased by non-changing genes. (6) Q-value calculation for *ClosestGene* scores.
(PDF)
