# Supplementary figures and images for: Assessing Computational Methods for Transcription Factor Target Gene Identification Based on ChIP-seq Data
Source: PLoS Comput Biol. 2013 Nov 21;9(11):e1003342. doi: 10.1371/journal.pcbi.1003342 (PMC3837635; doi:10.1371/journal.pcbi.1003342)

**A Perturbation (HemoChIP)**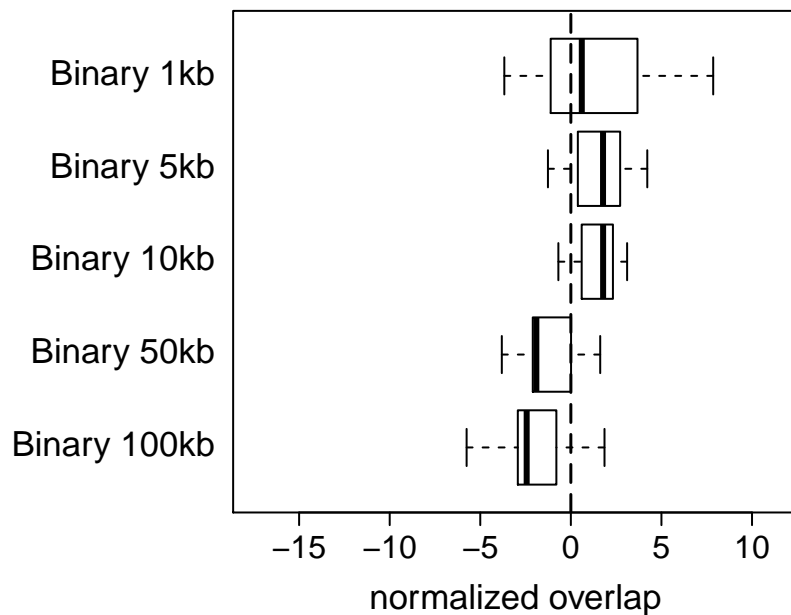**B Perturbation (ESChIP)**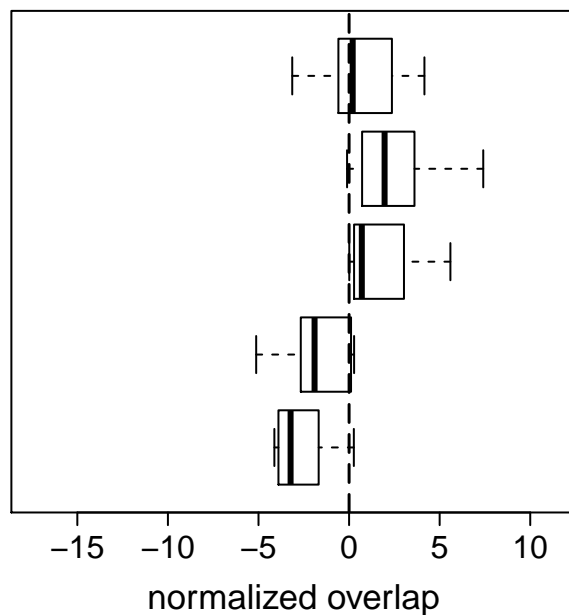**C Activity (HemoChIP)**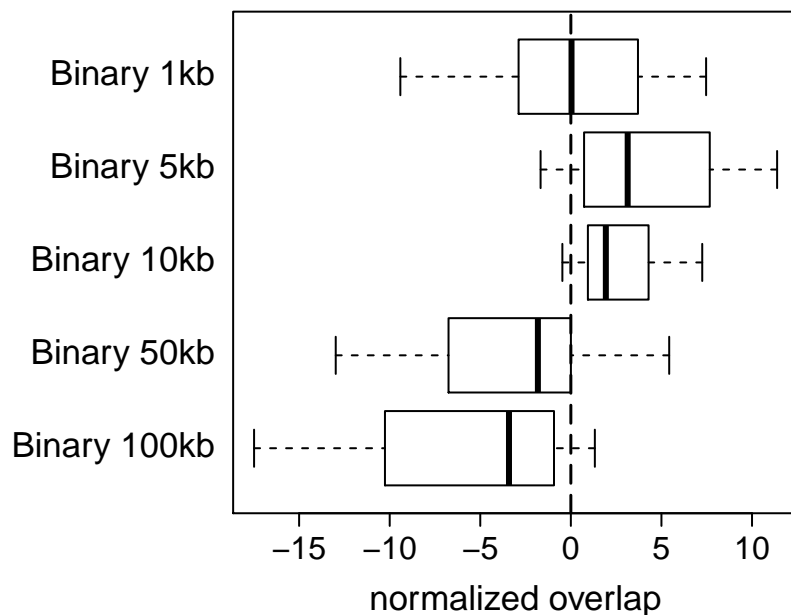**D Activity (ESChIP)**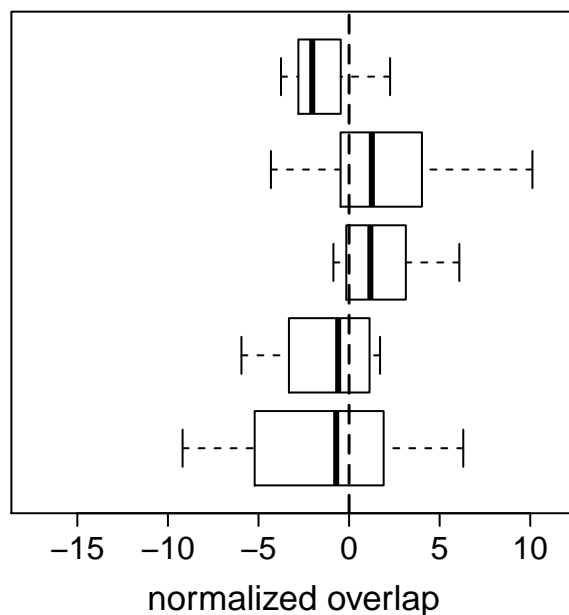

Supplement: Figure S1 — Performance of Binary for different window sizes. Overlap of the top 500 targets with the top 500 genes differentially expressed in (A) HemoChIP and (B) ESChIP TF perturbation experiments. Overlap of the top 500 targets with the top 500 genes differentially expressed (C) between erythroid and myeloid cells or (D) between undifferentiated (ES) and differentiated (MEF) cells. (PDF) [file pcbi.1003342.s001.pdf]

**A Perturbation (HemoChIP)**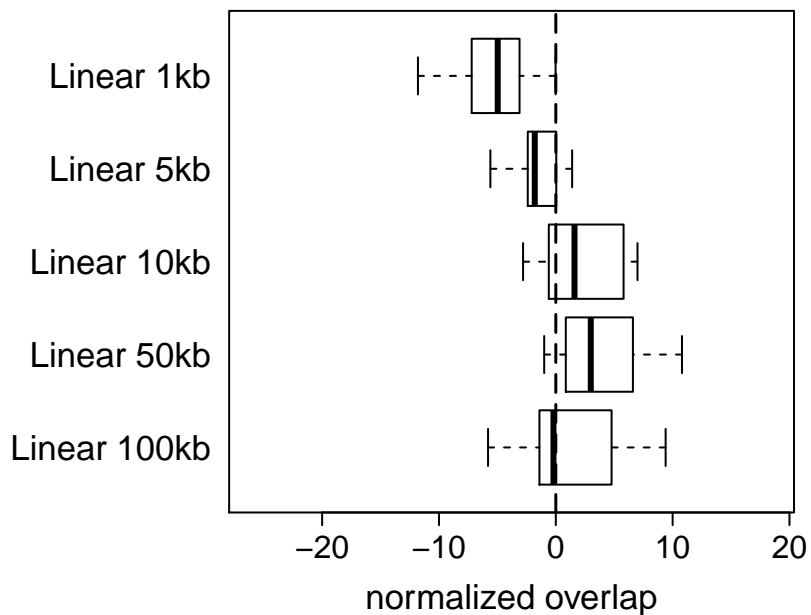**B Perturbation (ESChIP)**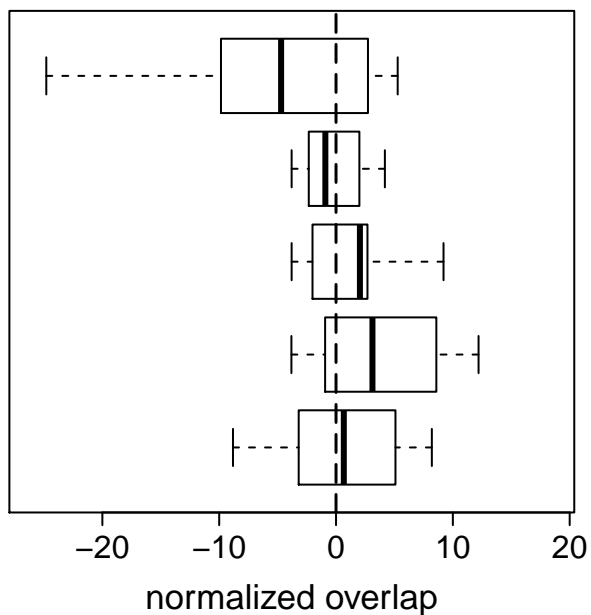**C Activity (HemoChIP)**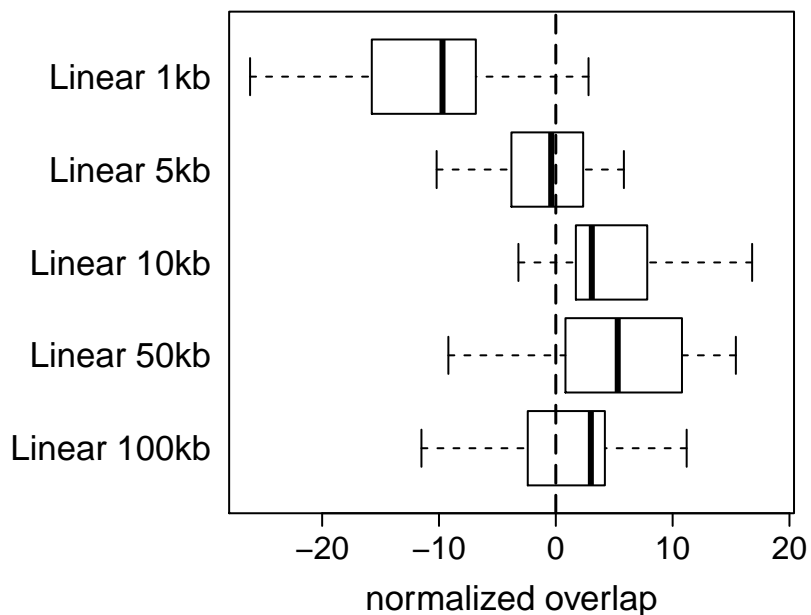**D Activity (ESChIP)**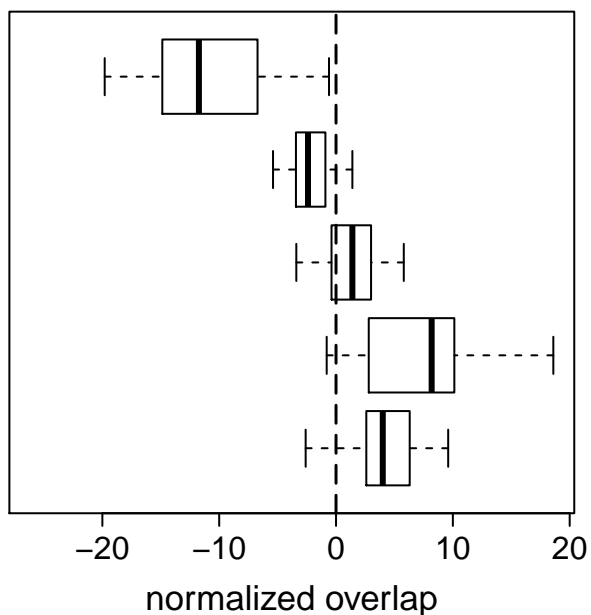

Supplement: Figure S2 — Performance of Linear for different window sizes. Overlap of the top 500 targets with the top 500 genes differentially expressed in (A) HemoChIP and (B) ESChIP TF perturbation experiments. Overlap of the top 500 targets with the top 500 genes differentially expressed (C) between erythroid and myeloid cells or (D) between undifferentiated (ES) and differentiated (MEF) cells. (PDF) [file pcbi.1003342.s002.pdf]

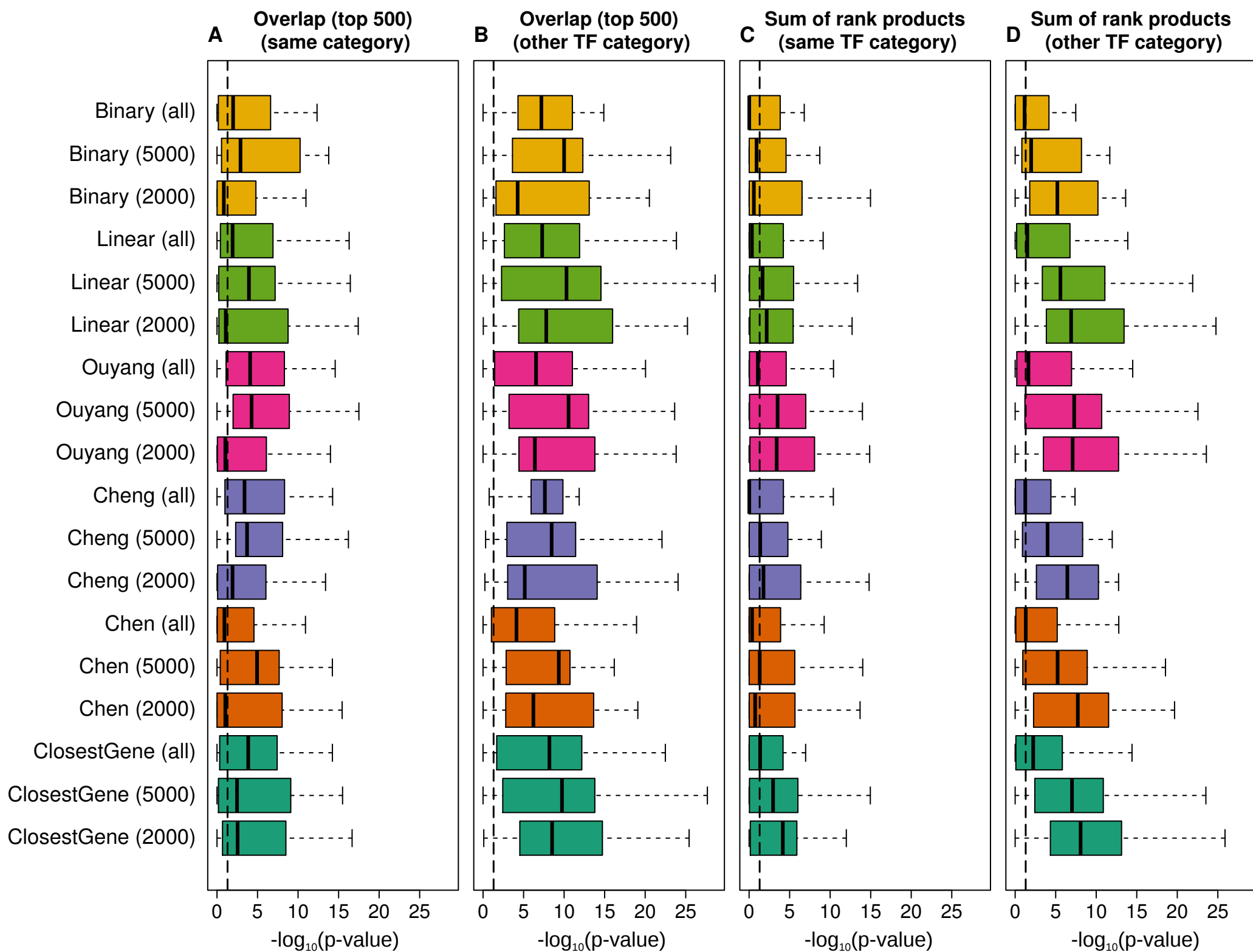

Supplement: Figure S3 — Specificity of TF-target prediction methods. High specificity of target predictions means that there should be higher congruence with perturbation expression data from the same factor than with perturbation data from different factors. Here, we compare measuring consistency based on the overlap of the top 500 genes (A, B; considering only top ranking genes) with measuring consistency based on the sum of rank products (C, D; comparing the entire rankings). Specificity is quantified as the difference between the scorings obtained for matching data (matching ChIP-seq and expression data) versus non-matching data, expressed as the –log10 (p–value) of the respective t-test (horizontal axis). ‘Same category’ (A, C) refers to comparing matching and non-matching pairings from the same cellular system. ‘Other category’ (B, D) refers to comparing matching pairings with pairing HemoChIP ChIP-seq data with ES cell expression data and vice versa. Larger differences are expected in the latter case. ‘All’ using all genes for the scoring; ‘5000’ and ‘2000’ using only the top 5000 (or 2000) most variable genes. The dashed line corresponds to p-value = 0.05. (PDF) [file pcbi.1003342.s003.pdf]

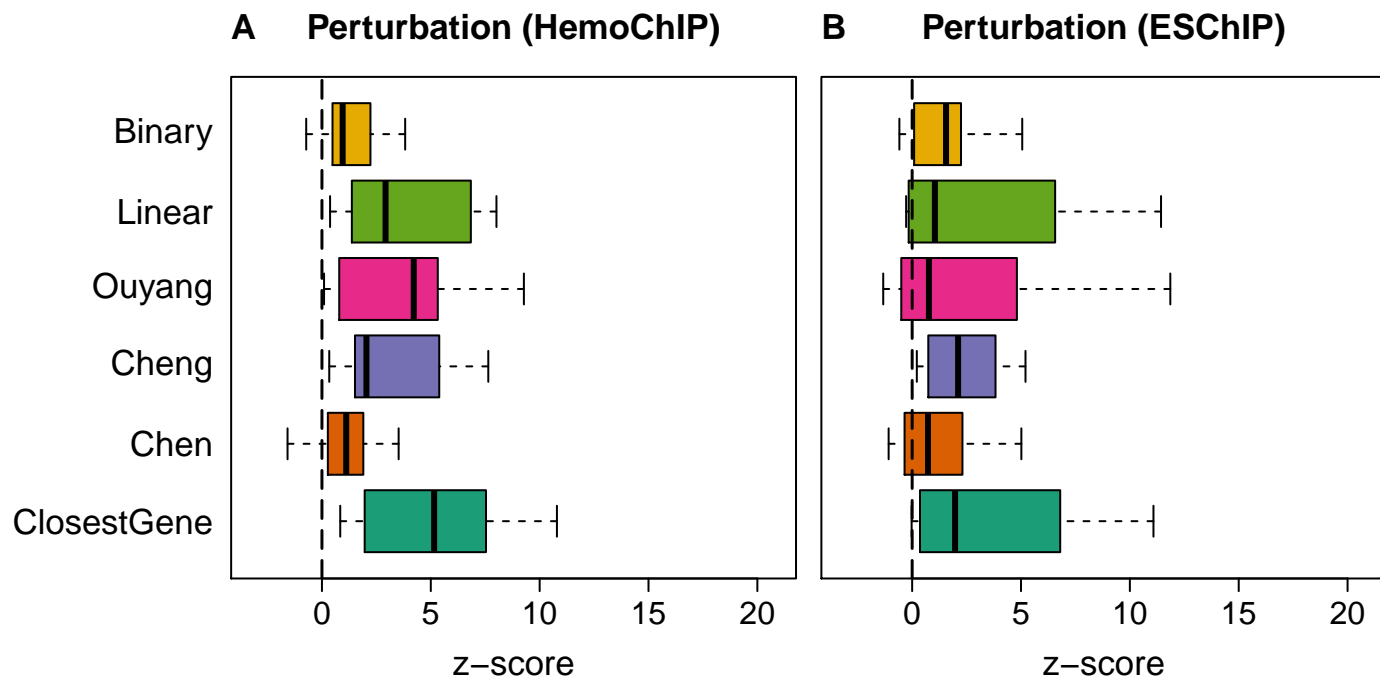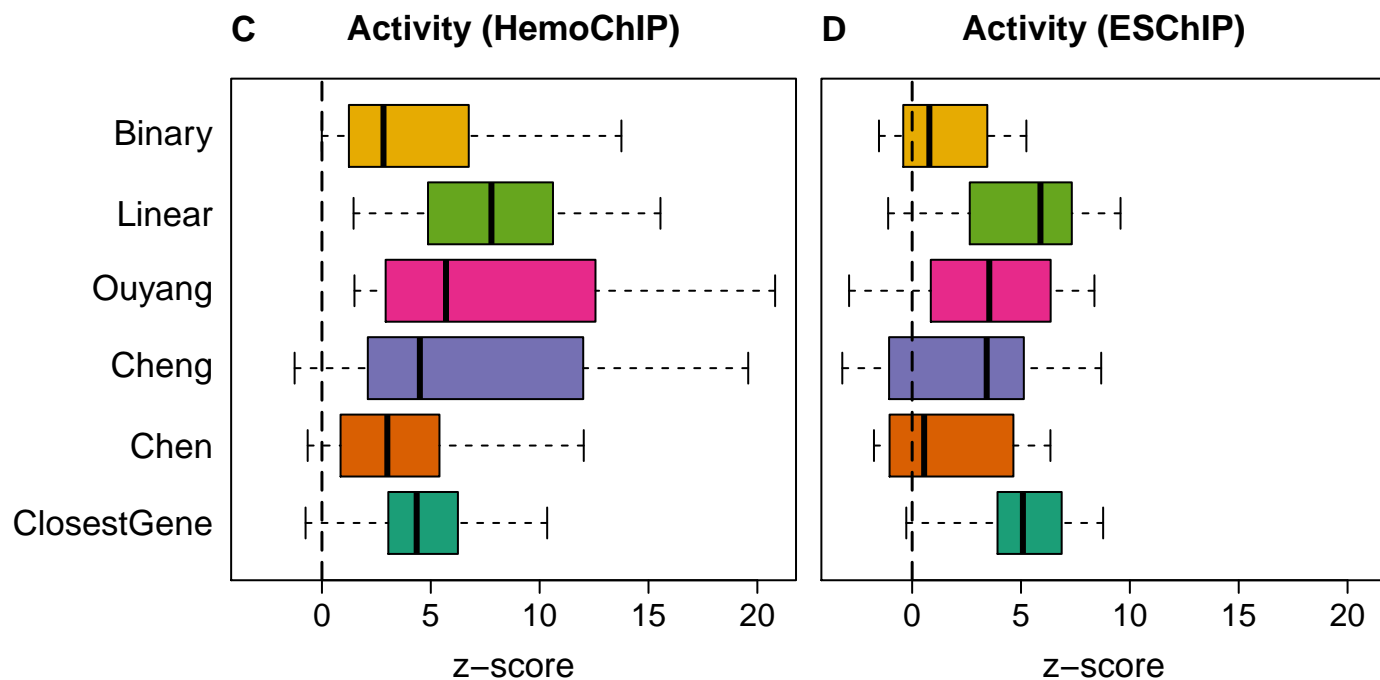

Supplement: Figure S4 — Significance of the targets recovery within top 500 genes. Z-scores of the overlap between the top 500 targets with the top 500 genes differentially expressed in (A) HemoChIP and (B) ESChIP TF perturbation experiments, (C) between erythroid and myeloid cells, (D) between undifferentiated (ES) and differentiated (MEF) cells. The difference between this visualization and Figure 2 of the main text is that here the overlaps are not normalized for the average performance across all methods. Thus, the z-scores do not account for the fact that some ChIP-seq studies intrinsically match better with the respective expression data (e.g. because identical cell types were used) than others. (PDF) [file pcbi.1003342.s004.pdf]

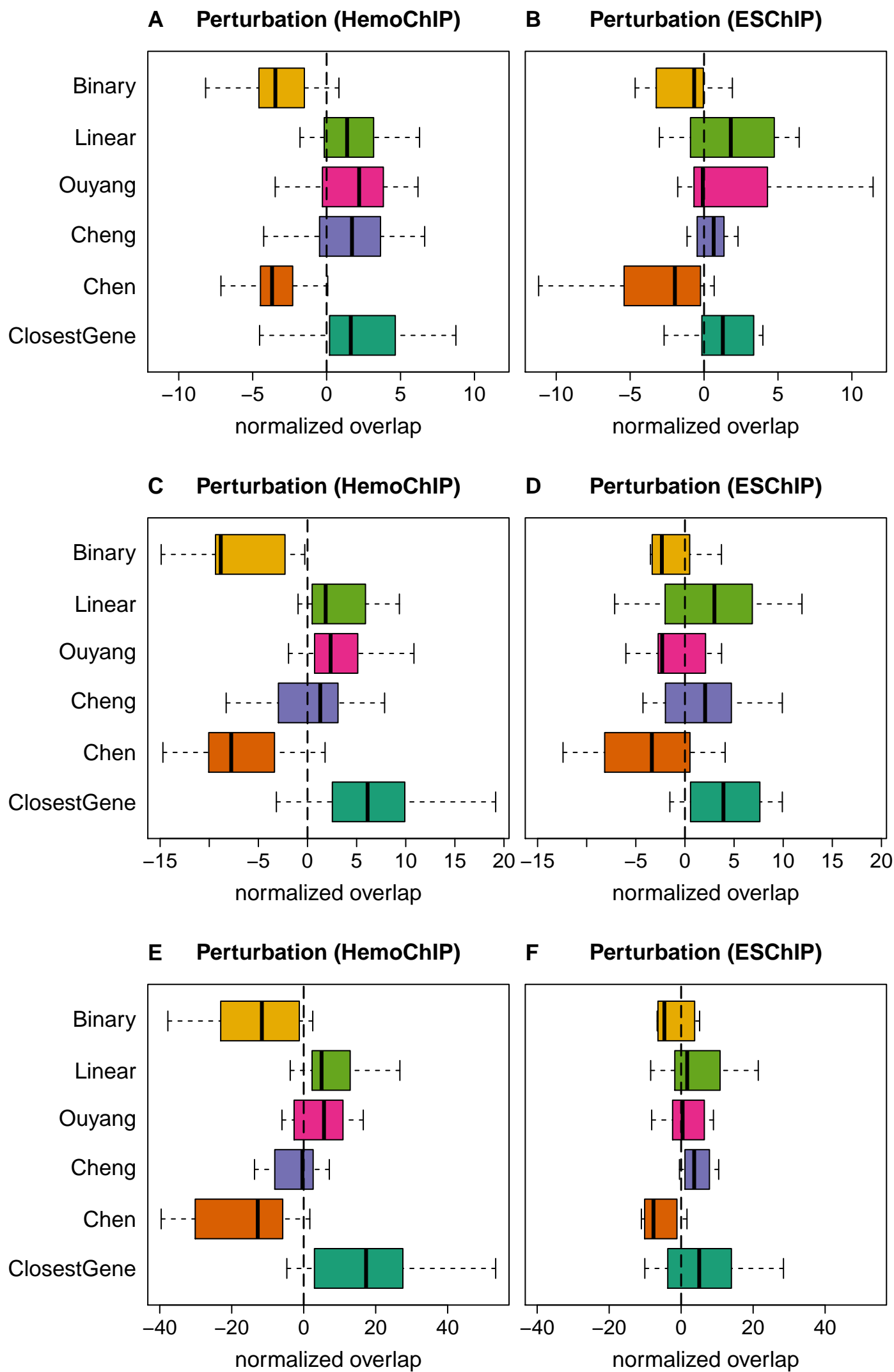

Supplement: Figure S5 — Targets recovery within different sets of genes (perturbation). Overlap of the top (A,B) 300, (C,D) 500 or (E,F) 1000 targets with the respective number of genes differentially expressed in (A,C,E) HemoChIP and (B,D,F) ESChIP TF perturbation experiments. (PDF) [file pcbi.1003342.s005.pdf]

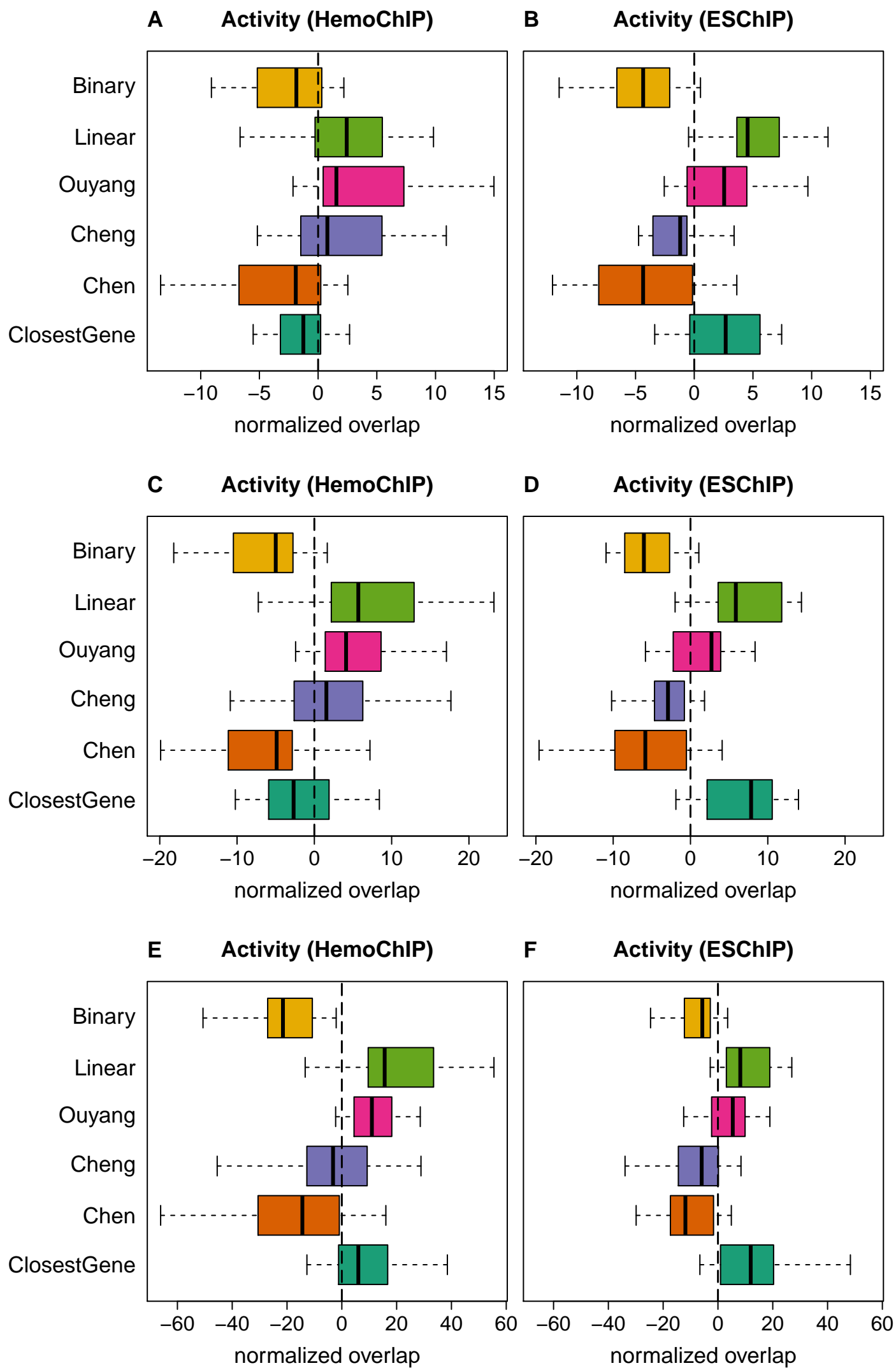

Supplement: Figure S6 — Targets recovery within different sets of genes (activity). Overlap of the top (A,B) 300, (C,D) 500 or (E,F) 1000 targets with the respective number of genes differentially expressed (A,C,E) between erythroid and myeloid cells or (B,D,F) between undifferentiated (ES) and differentiated (MEF) cells. (PDF) [file pcbi.1003342.s006.pdf]

**A HemoChIP**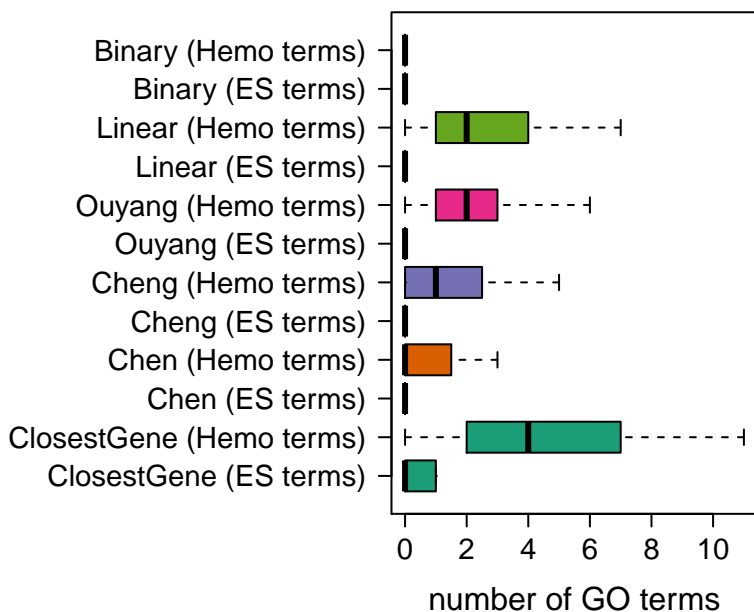**B ESChIP**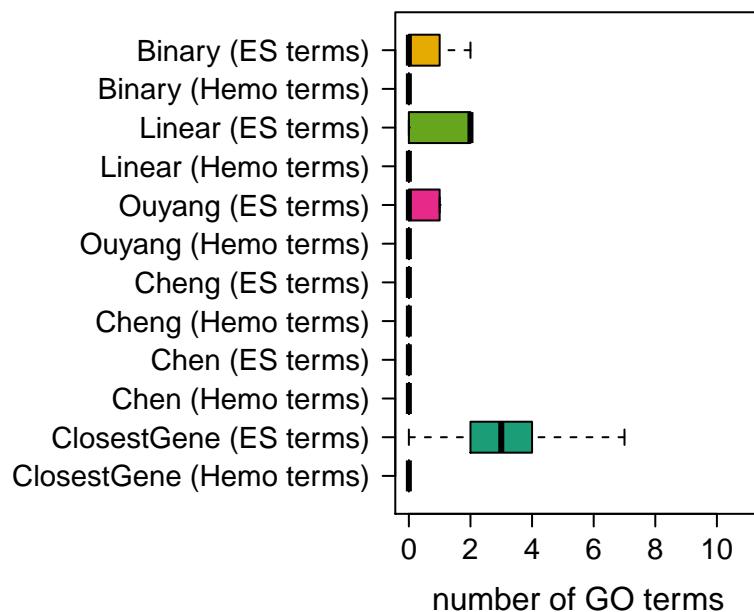**C HemoChIP**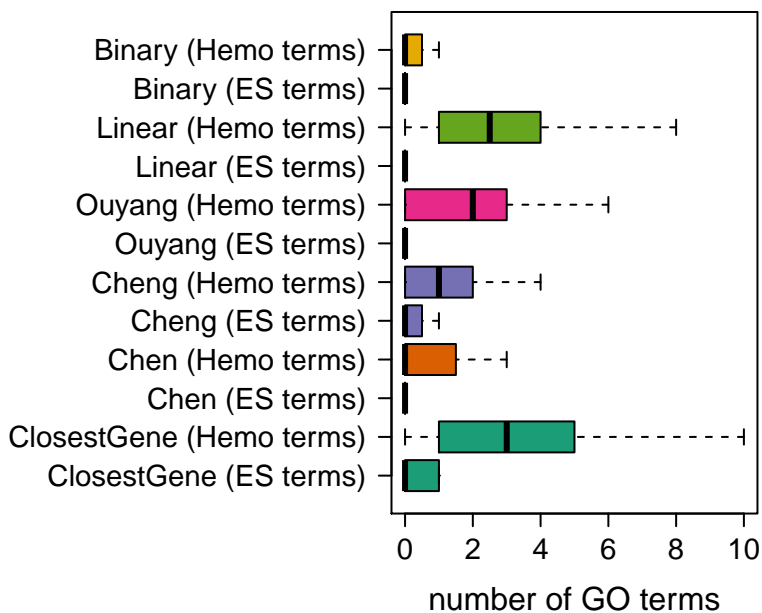**D ESChIP**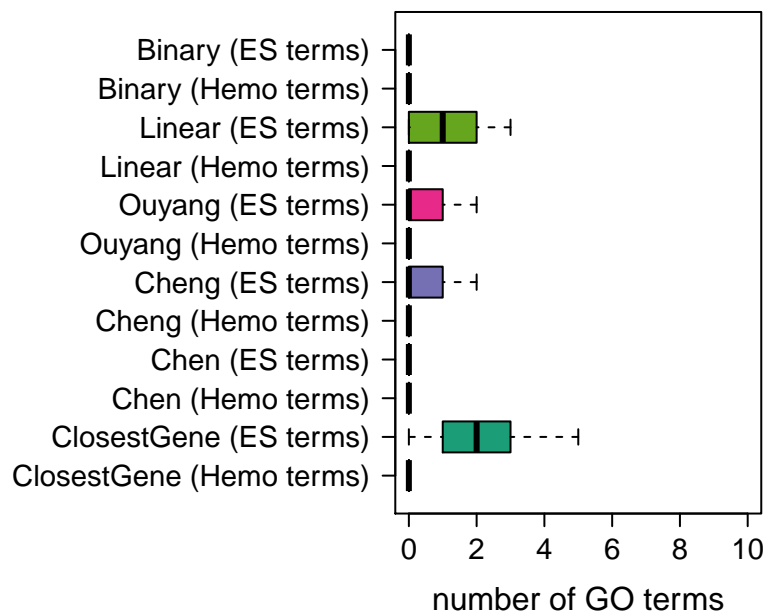**E HemoChIP**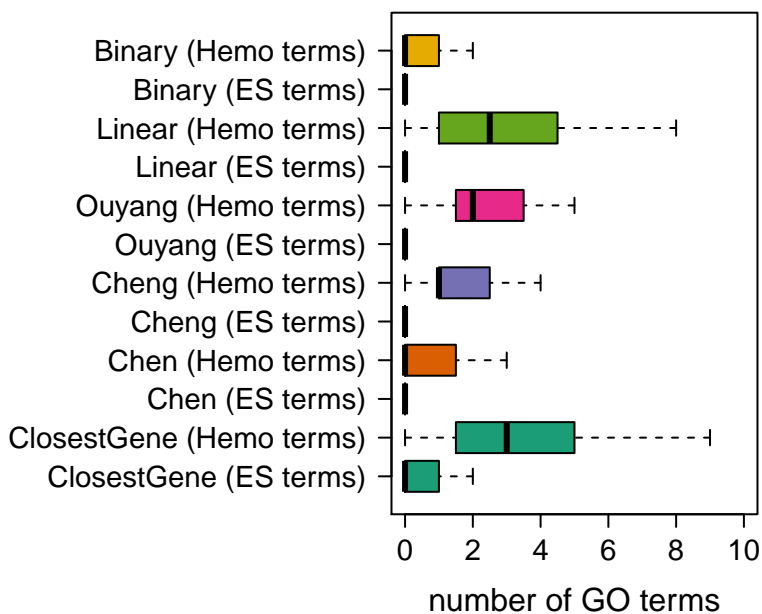**F ESChIP**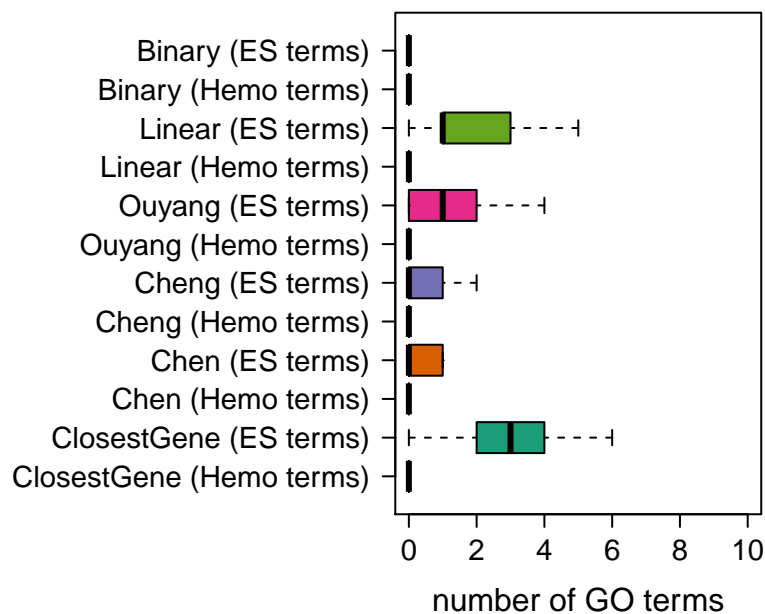

Supplement: Figure S7 — Functional homogeneity of targets for different sets of targets. Number of significantly enriched GO terms among top (A,B) 300, (C,D) 500 or (E,F) 1000 targets specific for a given cellular system and specific for the opposite cellular system for HemoChIP (A,C,E) and ESChIP (B,D,F). The specific terms are hematopoiesis or embryonic development related GO terms for HemoChIP and ESChIP, respectively. (PDF) [file pcbi.1003342.s007.pdf]

## A HemoChIP

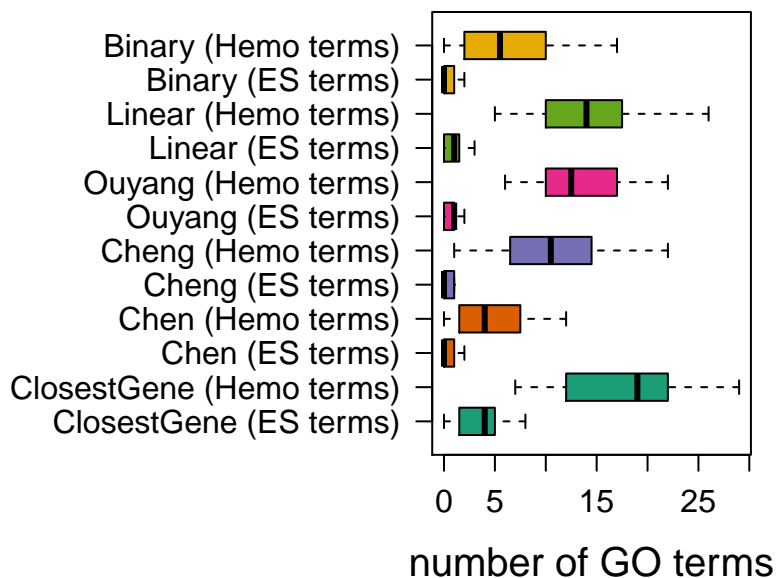

## B ESChIP

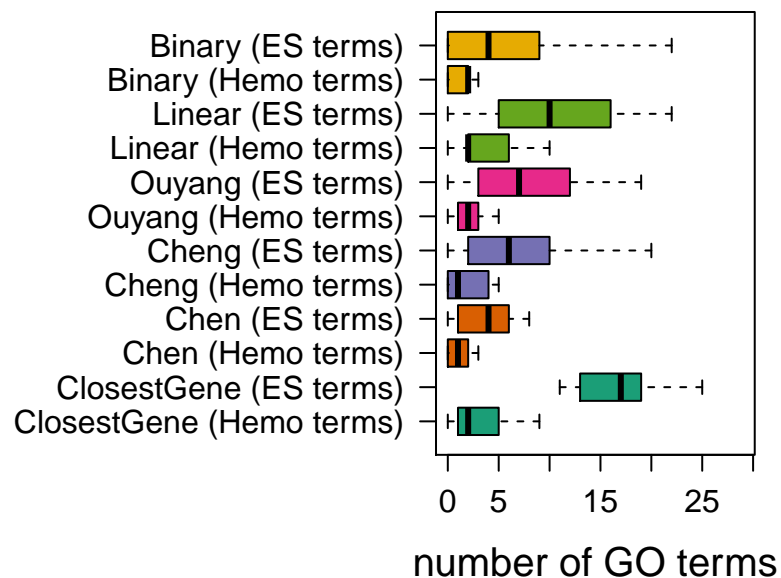

## C HemoChIP

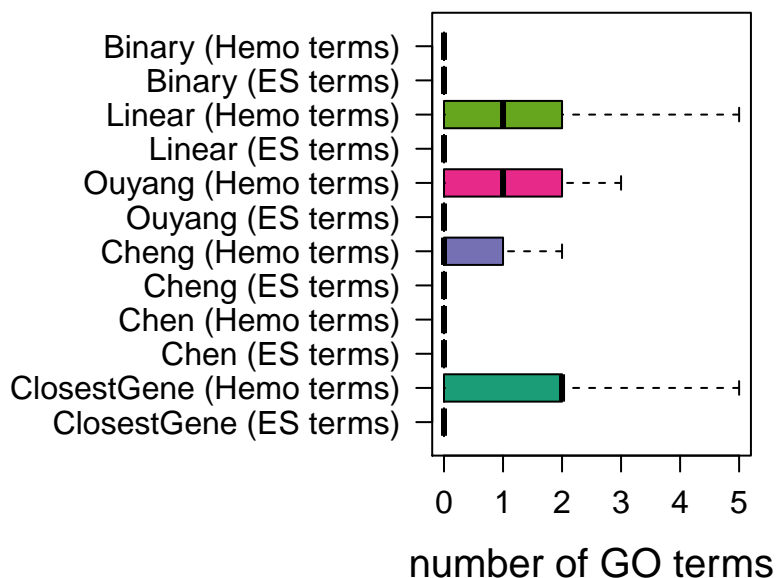

## D ESChIP

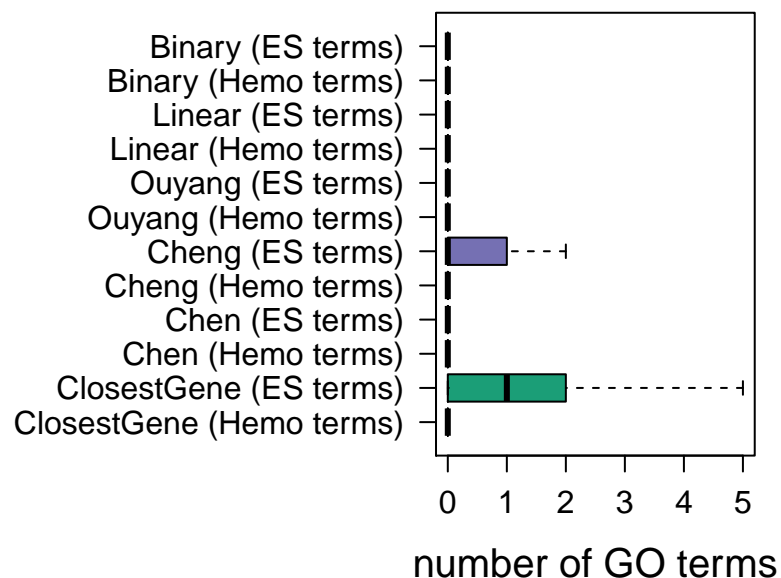

Supplement: Figure S8 — Functional homogeneity of the targets for different enrichment significance thresholds. Number of significantly enriched GO terms specific for a given cellular system and specific for the opposite cellular system for HemoChIP (A,C) and ESChIP (B,D). The significantly enriched GO terms were defined as the ones with p–value<0.05 (A,B) or p–value<0.0001 (C,D) of the Fisher's exact test. (PDF) [file pcbi.1003342.s008.pdf]

A

B

C

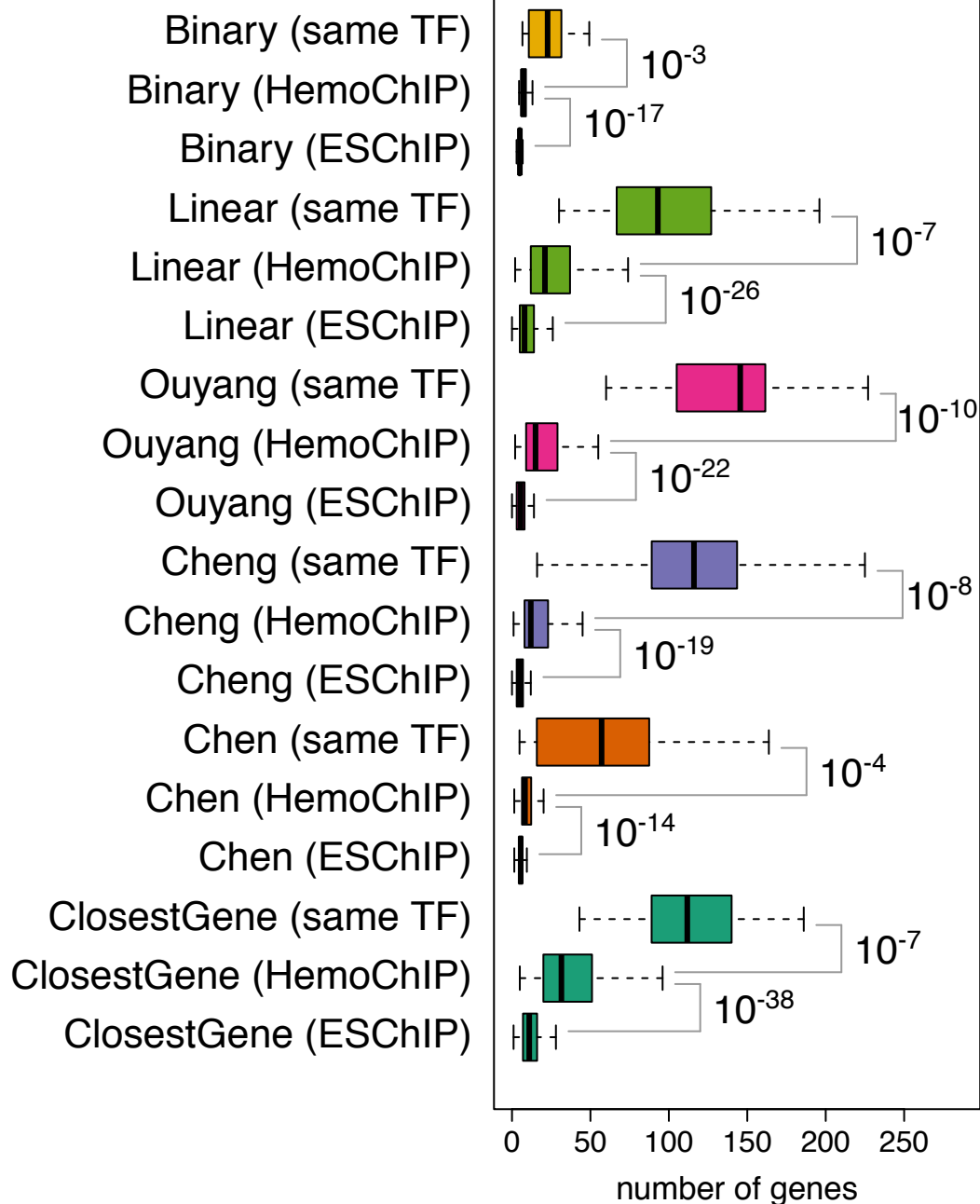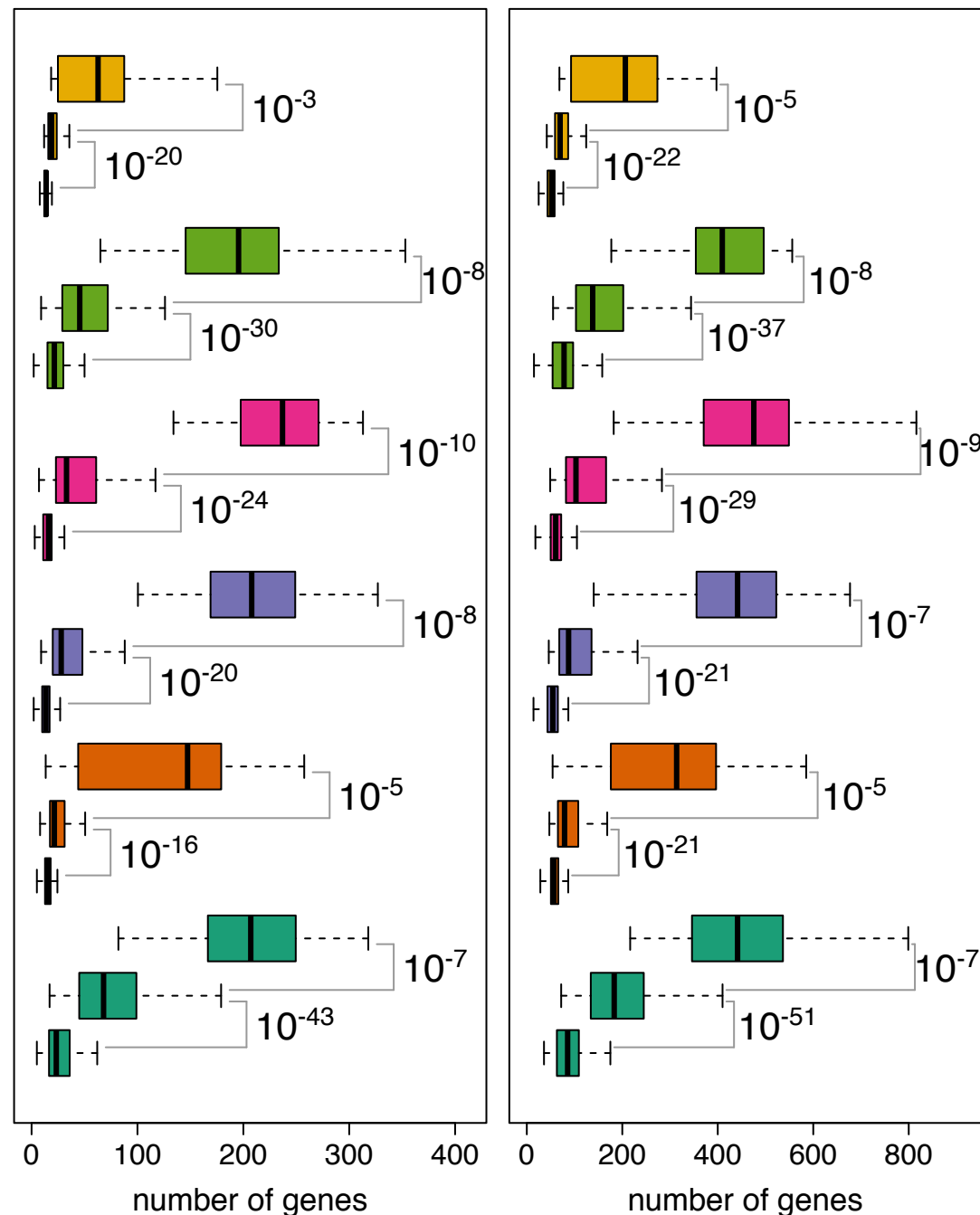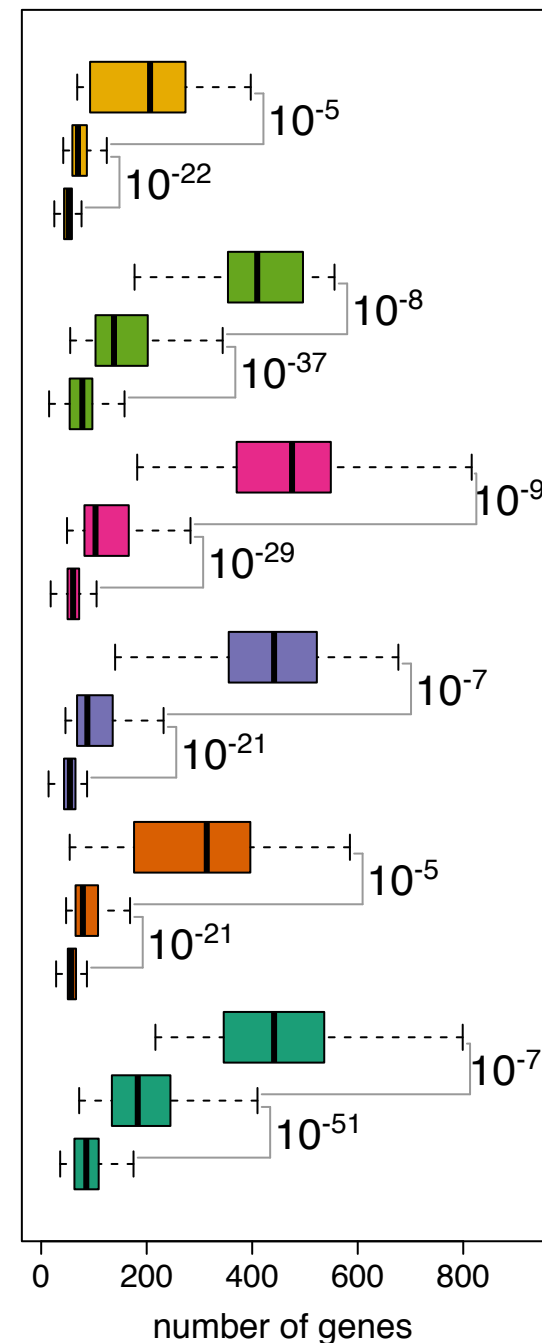

Supplement: Figure S9 — Consistency of target gene predictions for different sets of targets. Independent ChIP-seq experiments are available for some of the factors measured in the hematopoietic system. Consistency of target predictions is quantified as the overlap between the top (A) 300, (B) 500 or (C) 1000 target genes. Results are summarized based on intersecting targets from pairs of ChIP-seq experiments measuring the same transcription factor (‘same TF’), using ChIP-seq experiments from different factors, but the same system (hematopoietic cells, ‘HemoChIP’) and using ChIP-seq experiments from a different system (ES cells, ‘ESChIP’). Numbers on the right are rounded p-values, measuring the significance of the difference between the overlaps (t-test). P-values for the comparison ‘same TF’ versus ‘HemoChIP’ are generally less significant than ‘HemoChIP’ versus ‘ESChIP’, because the number of comparisons (observations) is smaller. (PDF) [file pcbi.1003342.s009.pdf]

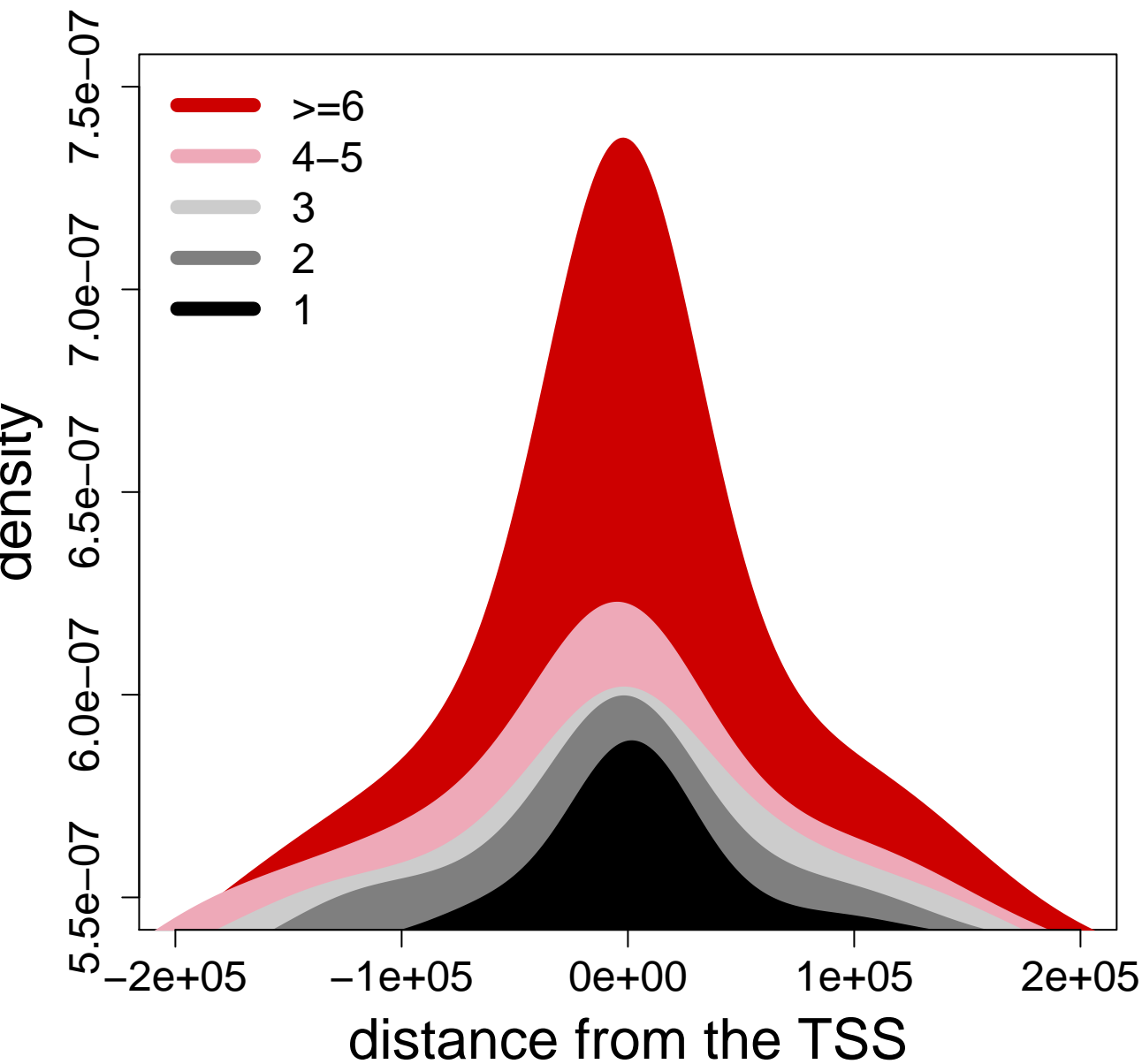

Supplement: Figure S10 — Characterization of binding events with different numbers of co-factors. Density distribution of binding sites around TSSs as a function of the number of factors binding (as shown in legend). Figure shows a fragment of the density distribution plot; the density below the lines sums to 1 for distances from −1 Mb to 1 Mb from the TSS. (PDF) [file pcbi.1003342.s010.pdf]

**A**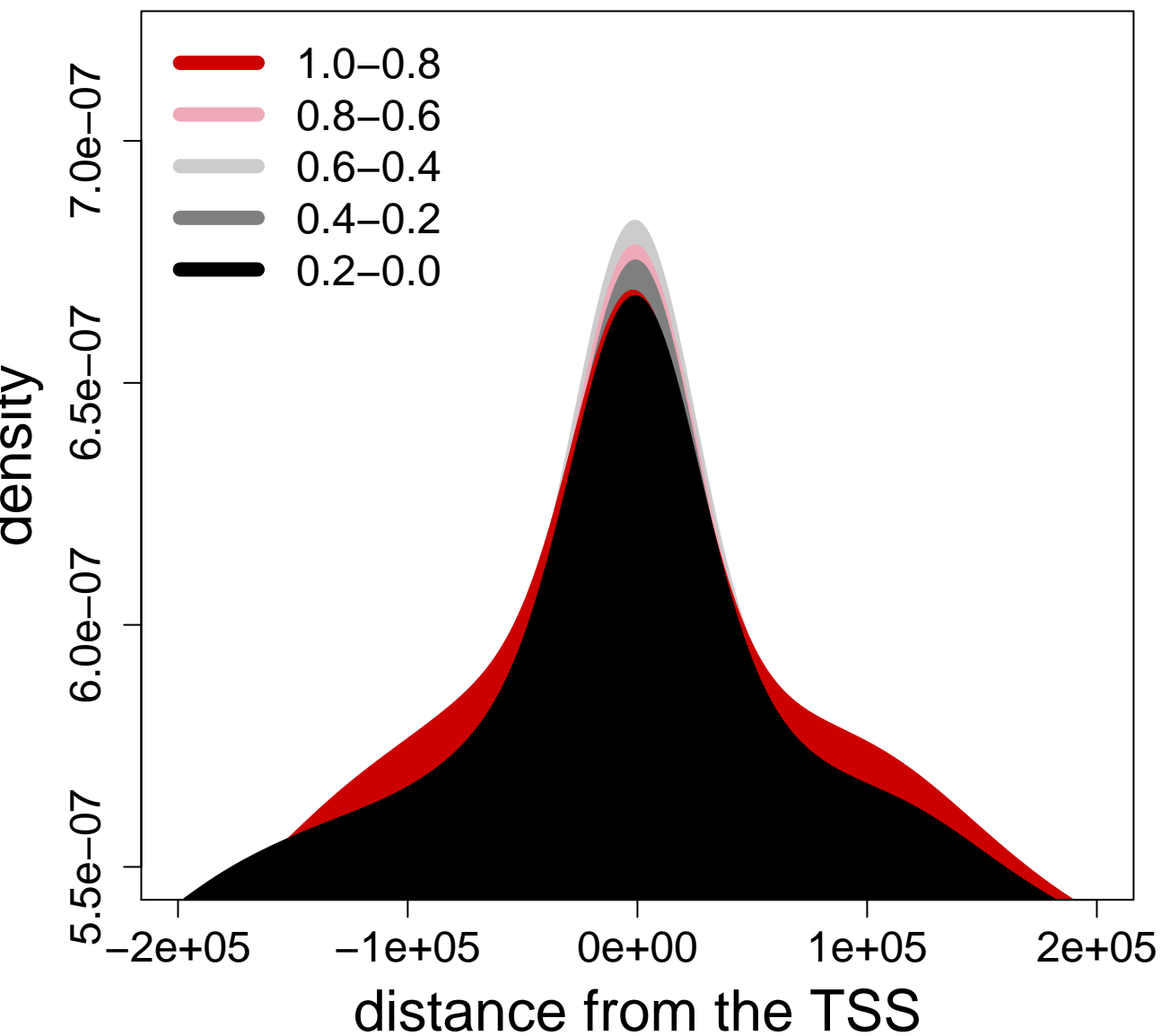**B**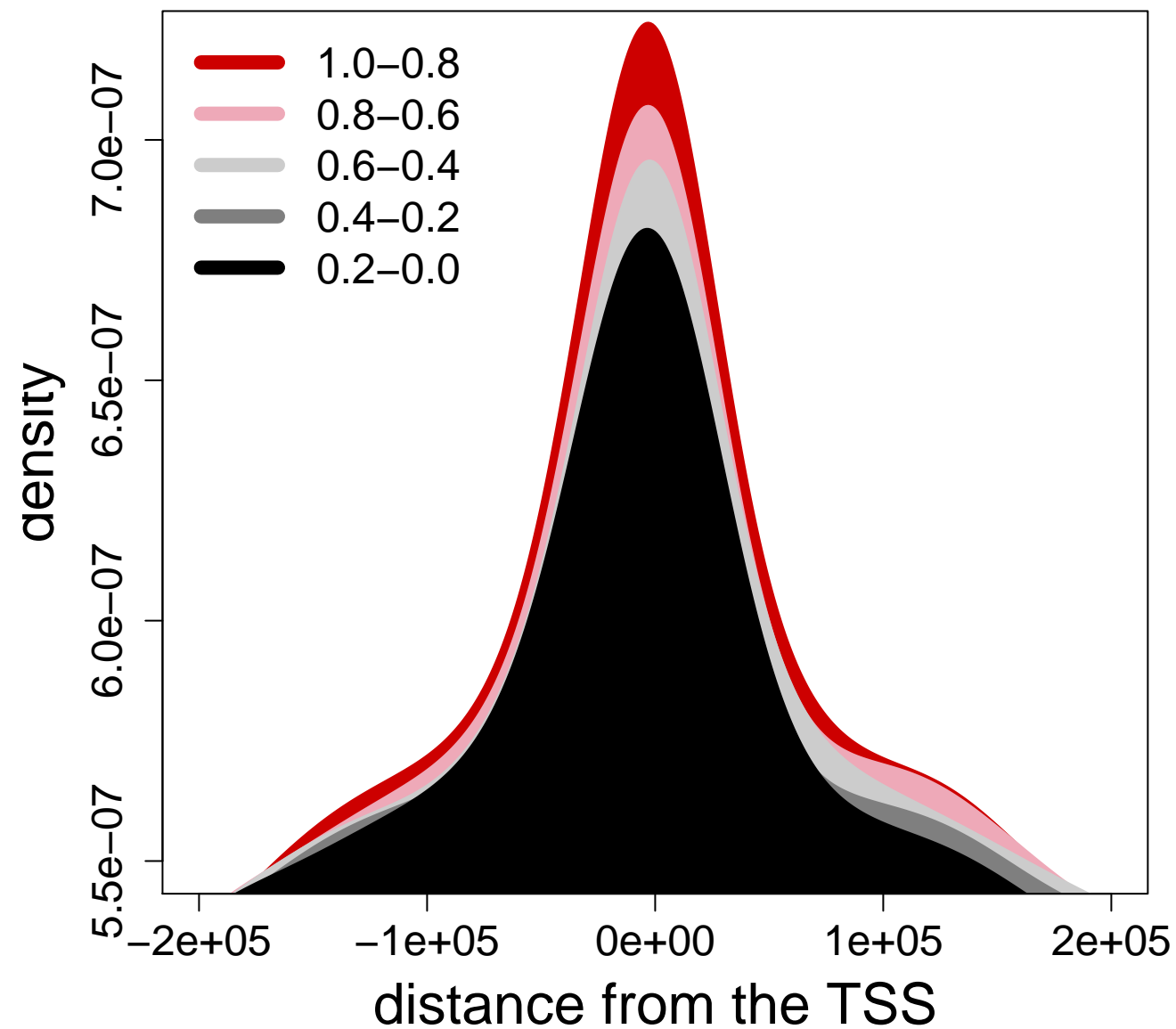

Supplement: Figure S11 — Characterization of binding events with different intensities. Density distribution of peaks around TSSs as a function of normalized peak intensity (1 = highest intensity, 0 = lowest intensity; see legend) for (A) HemoChIP and (B) ESChIP. Figure shows fragments of density distribution plots; the below the density lines sums to 1 for distances from −1 Mb to 1 Mb from the TSS. (PDF) [file pcbi.1003342.s011.pdf]

**A Perturbation (HemoChIP)**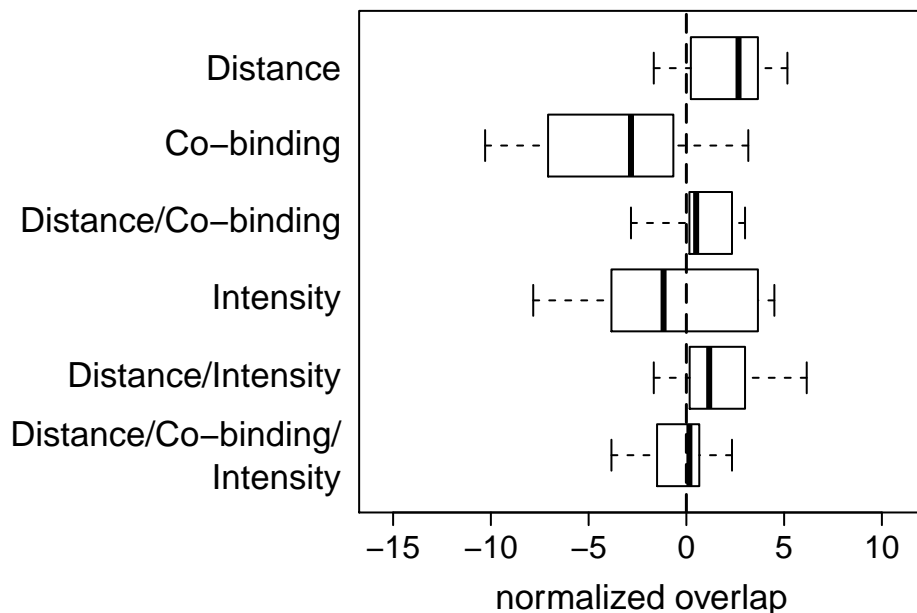**B Perturbation (ESChIP)**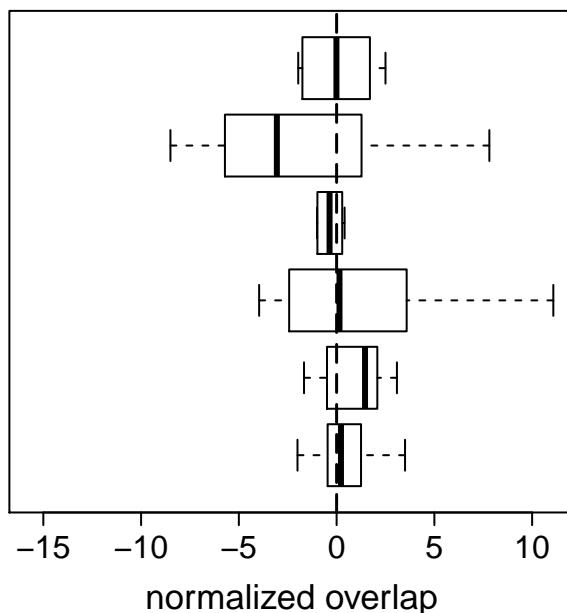**C Activity (HemoChIP)**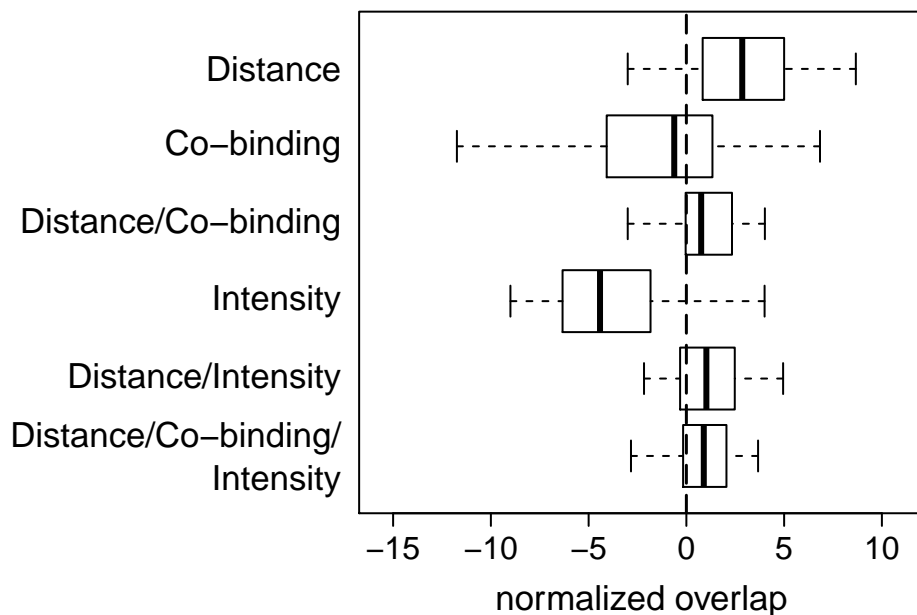**D Activity (ESChIP)**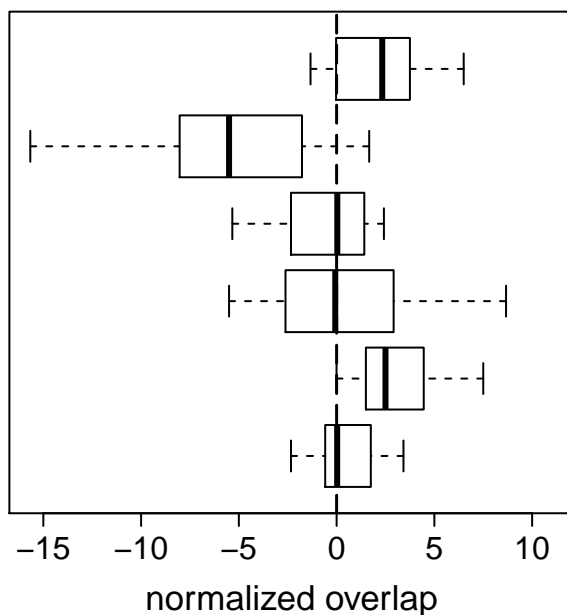

Supplement: Figure S12 — Combination of peak-scoring criteria. Peaks are scored based on their distance to the TSS (Distance), intensity (Intensity) or number of co-binding factors at the same site (Co-binding) or combinations thereof (as indicated). Peak-to-gene assignment and score integration is done using the ClosestGene scheme. Overlap of the top 500 targets with the top 500 genes differentially expressed in (A) HemoChIP and (B) ESChIP TF perturbation experiments. Overlap of the top 500 targets with the top 500 genes differentially expressed (C) between erythroid and myeloid cells or (D) between undifferentiated (ES) and differentiated (MEF) cells. (PDF) [file pcbi.1003342.s012.pdf]

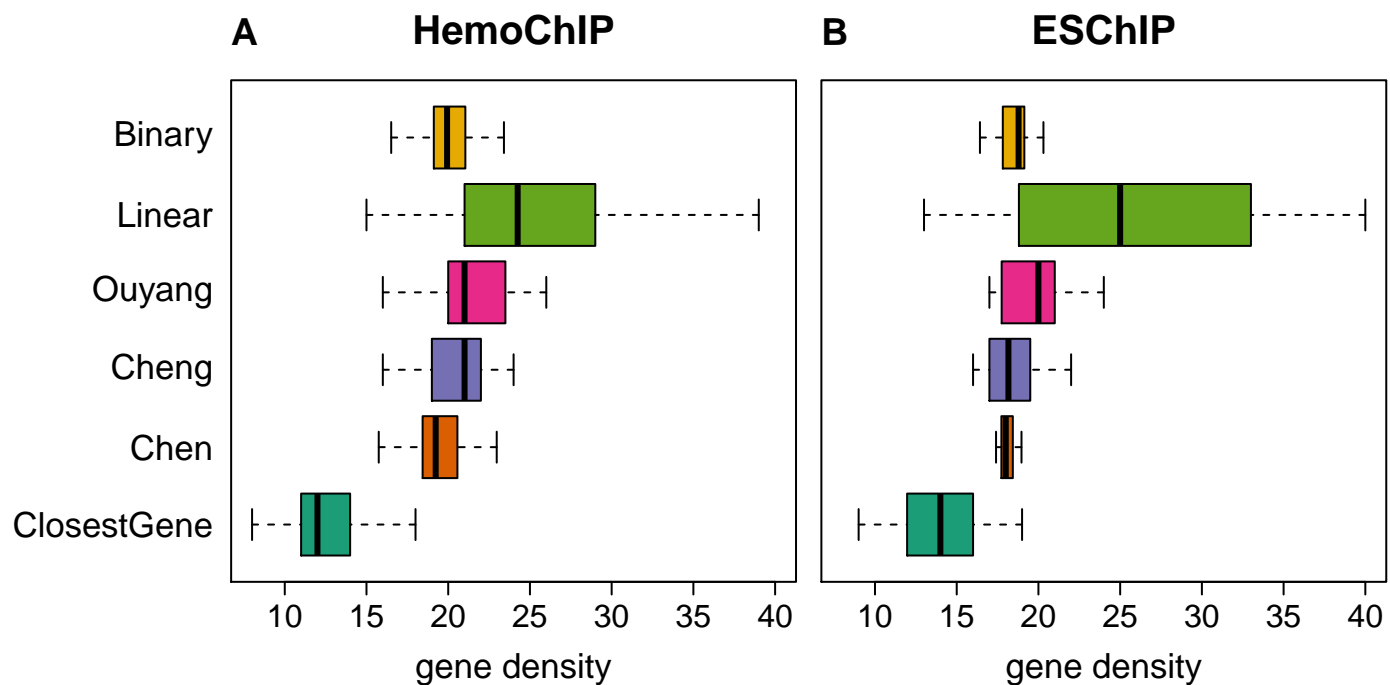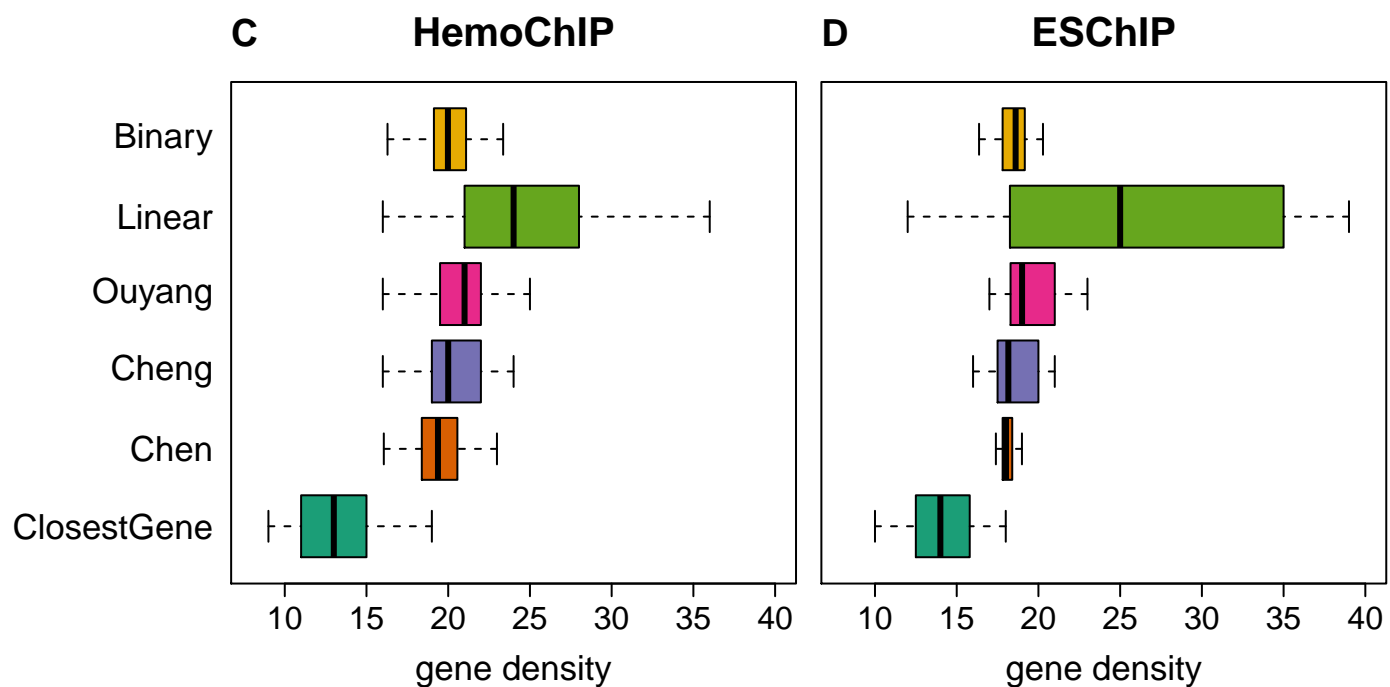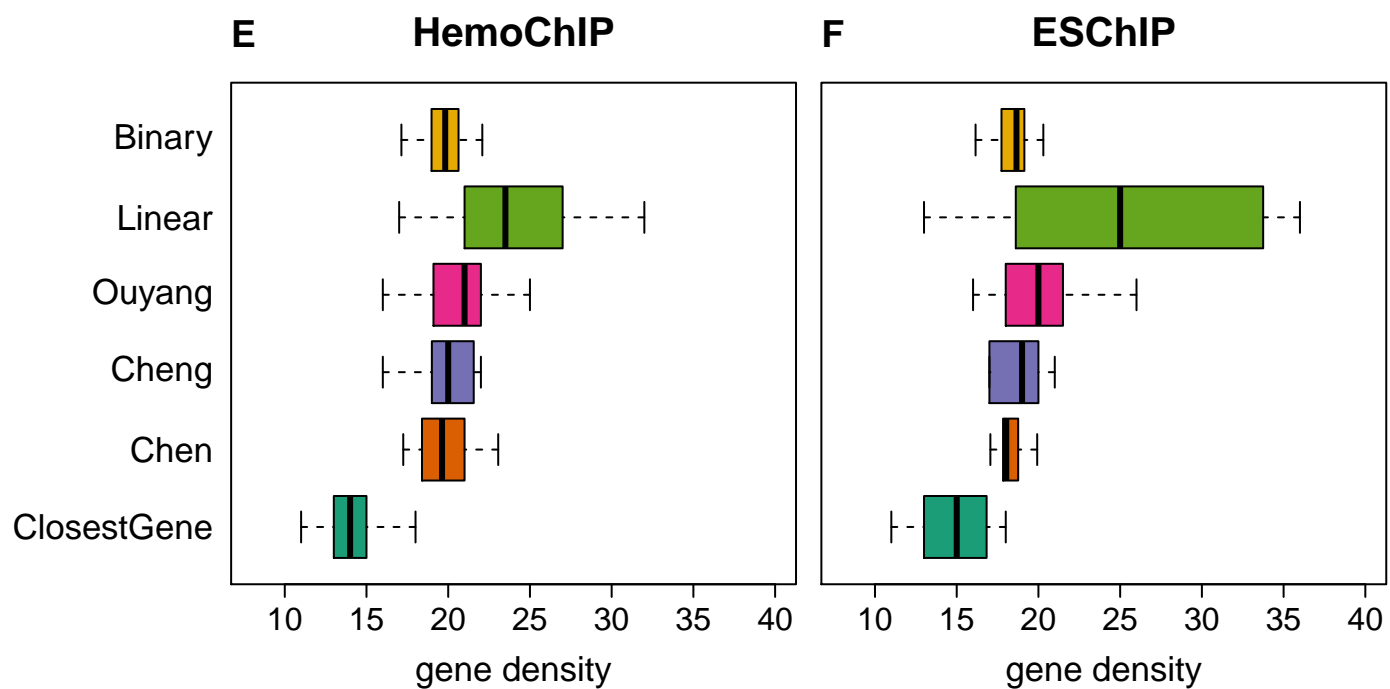

Supplement: Figure S13 — Gene density in target regions for different sets of targets. Gene density (number of genes inside 1 Mb regions around the target gene's TSS) of the regions harboring the top 300 (A, B), 500 (C, D) or 1000 (E, F) genes across the studies in the HemoChIP (A, C, E) and ESChIP datasets (B, D, F). (PDF) [file pcbi.1003342.s013.pdf]

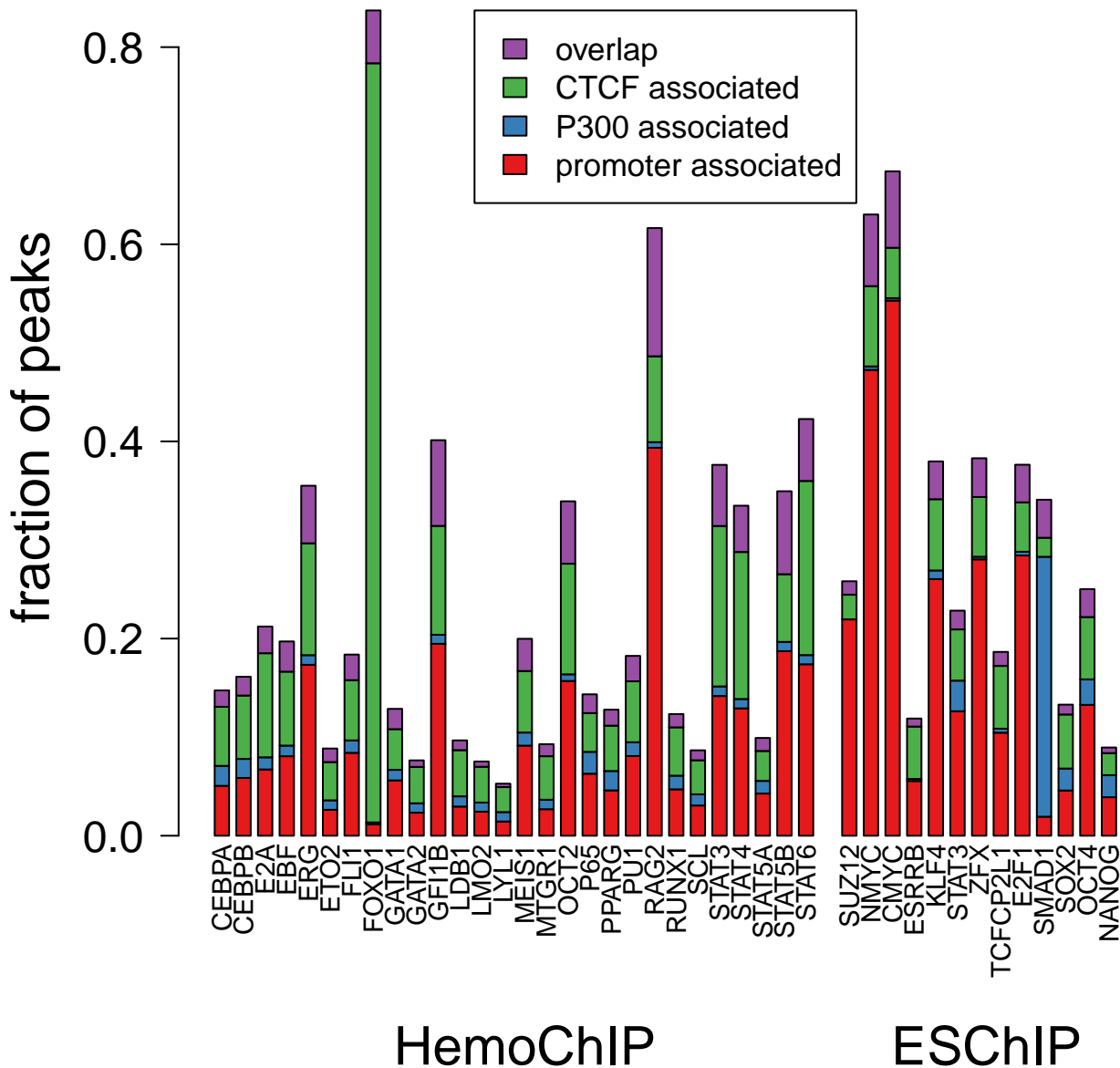

Supplement: Figure S14 — Characterization of transcriptional regulators. Fraction of peaks binding at promoters, or co-occurring with P300 or CTCF binding. Overlap encompasses peaks falling in more than one of those categories. Regulators are grouped depending on the cellular system in which the corresponding ChIP-seq study has been conducted (hematopoietic cells or embryonic stem cells, respectively). (PDF) [file pcbi.1003342.s014.pdf]

**A Perturbation (HemoChIP)**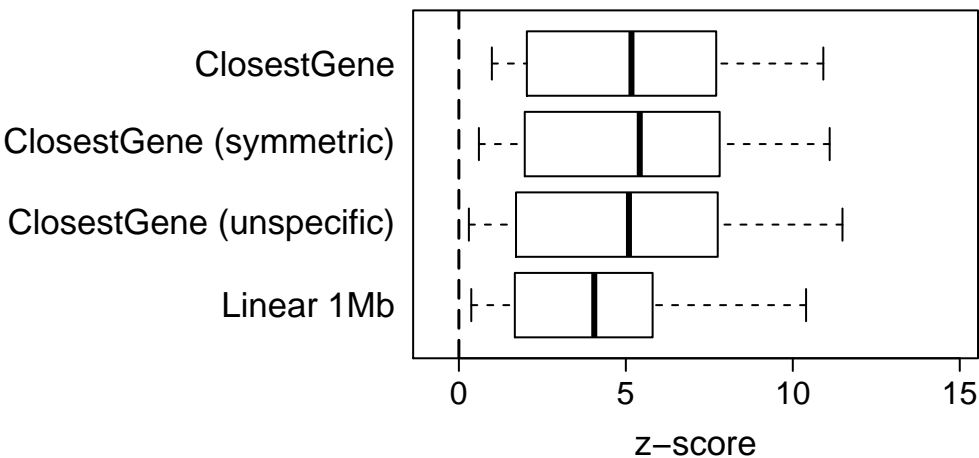**B Perturbation (ESChIP)**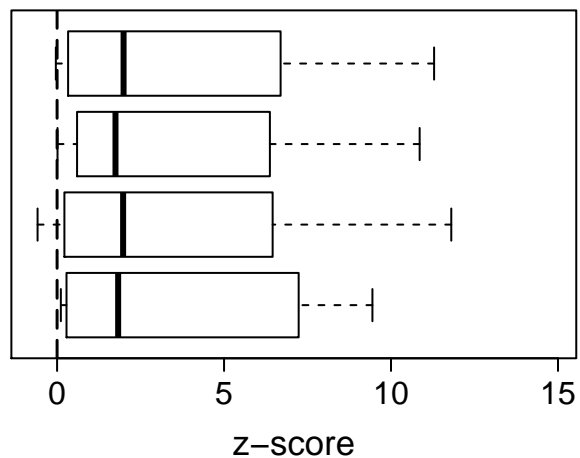**C Activity (HemoChIP)**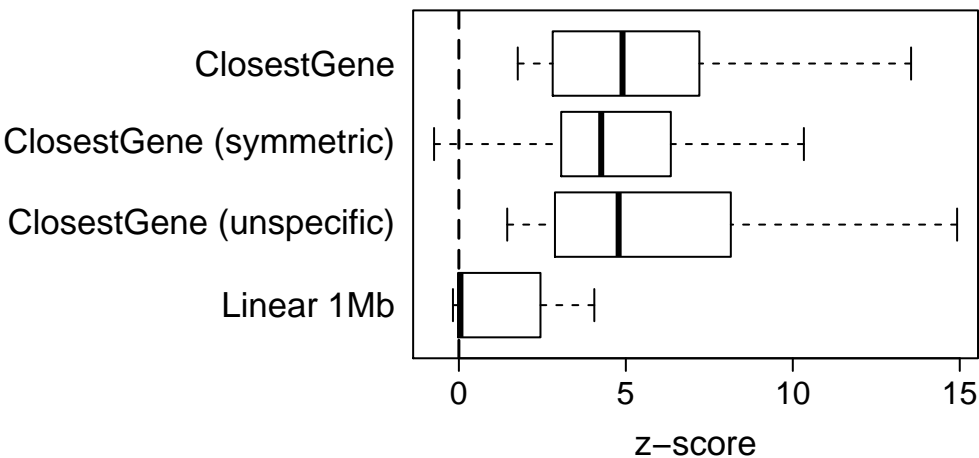**D Activity (ESChIP)**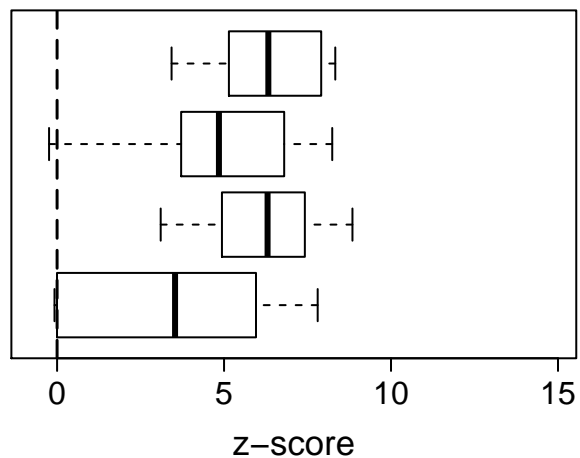

Supplement: Figure S15 — Performance of ClosestGene for different scorings. Z-scores of the overlap between the top 500 targets with the top 500 genes differentially expressed in (A) HemoChIP and (B) ESChIP TF perturbation experiments, (C) between erythroid and myeloid cells, (D) between undifferentiated (ES) and differentiated (MEF) cells. ClosestGene corresponds to ClosestGene using TF-specific peak-to-gene distance distribution. ClosestGene (symmetric) corresponds to the variant where a distribution symmetric around a TSS (obtained by pooling all peak-to-gene distances without distinguishing between upstream and downstream peaks). ClosestGene (unspecific) corresponds to the variant where a distribution specific for another TF is used for peak scoring. Linear 1Mb corresponds to the variant where peaks assigned to the TSS are scored using linearly decreasing weights. (PDF) [file pcbi.1003342.s015.pdf]

**A** Oct4 (ESChIP)

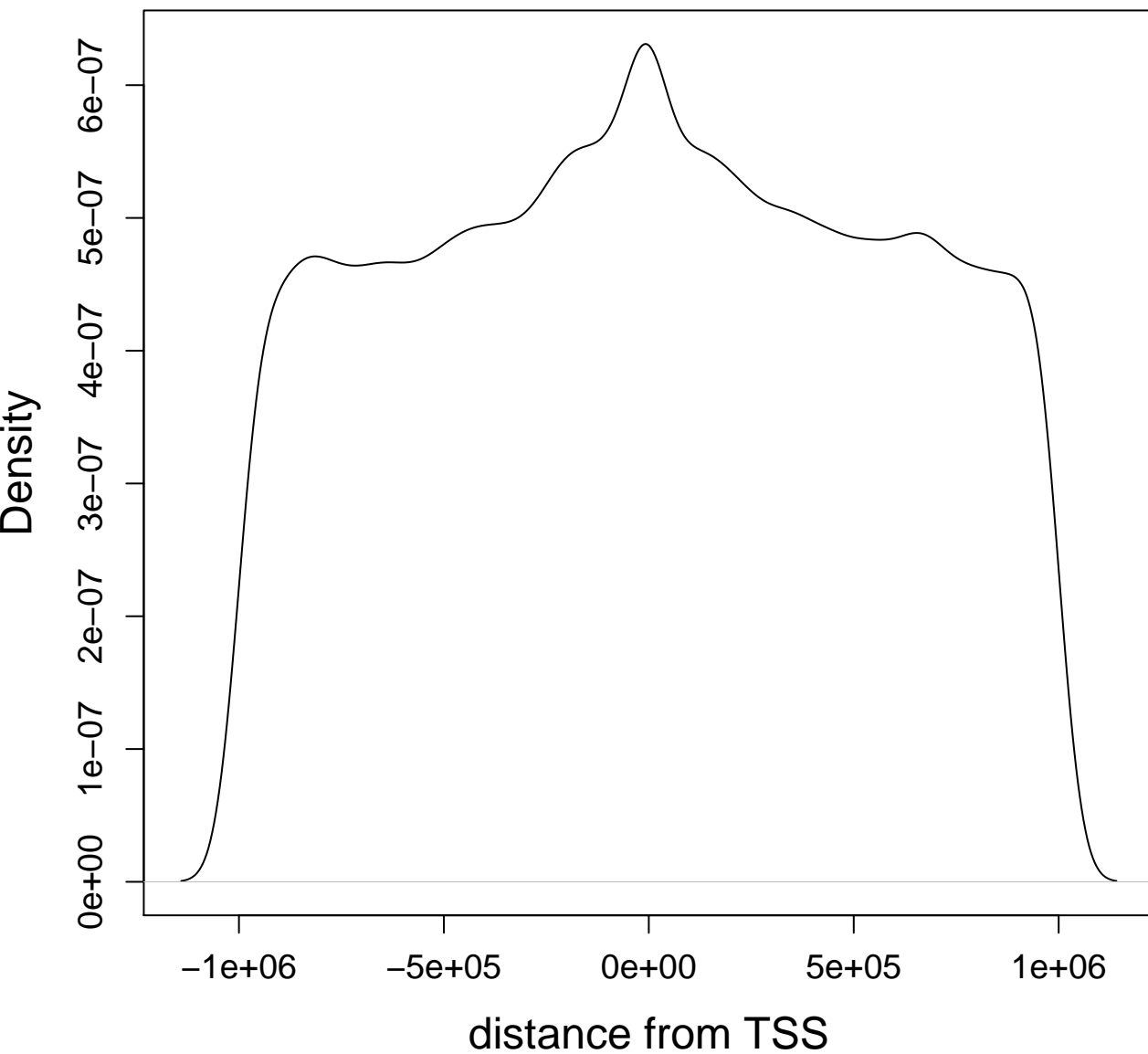

**B** P300 (ESChIP)

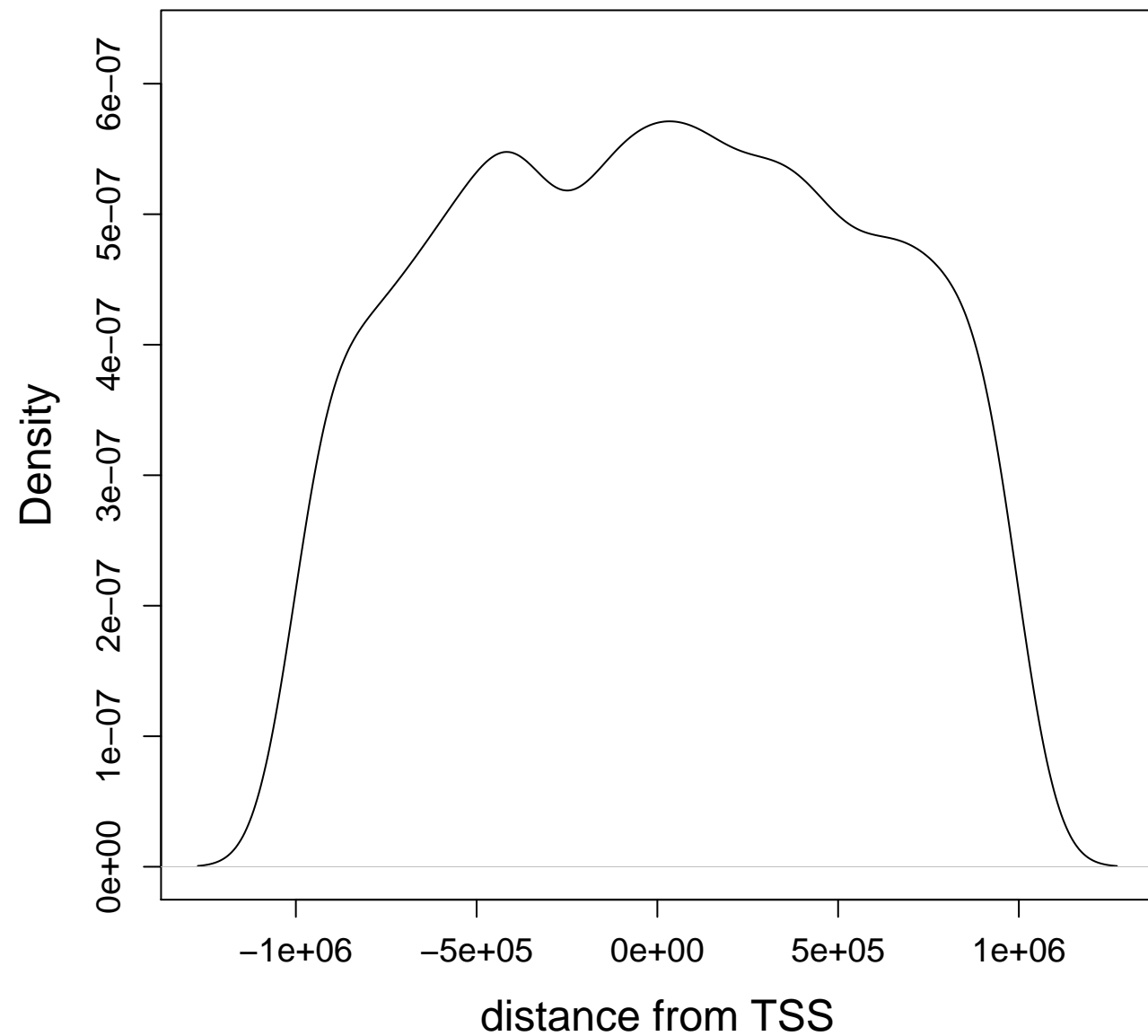

Supplement: Figure S16 — Peak-to-gene distance distribution. Peak-to-gene distance distribution for (A) OCT4 and (B) P300 used for peak scoring. (PDF) [file pcbi.1003342.s016.pdf]

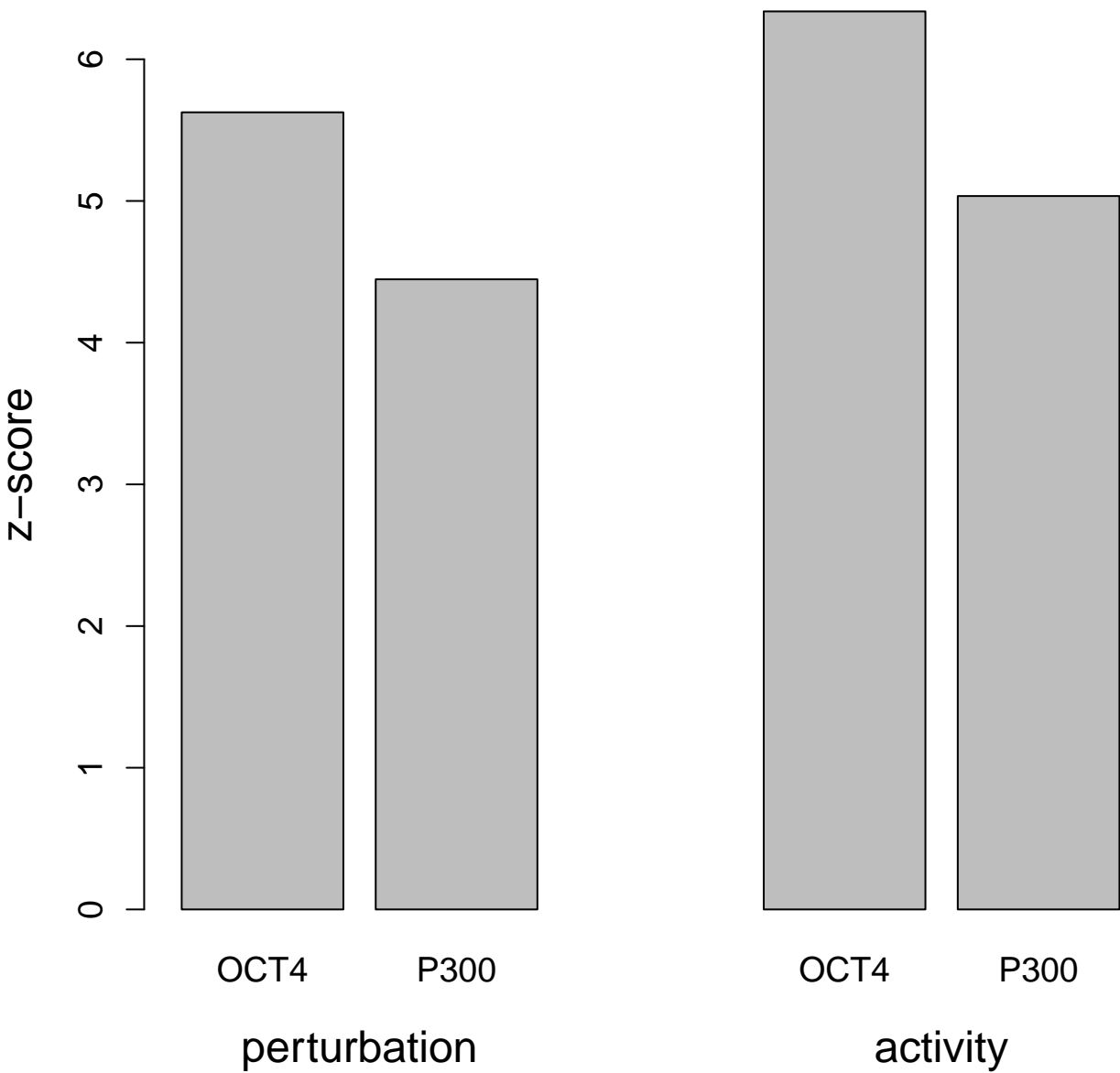

Supplement: Figure S17 — Performance of the OCT4 target prediction using different distributions. Z-score representing the significance of the overlap between top 500 targets and top 500 genes differentially expressed after Oct4 knock-down (‘perturbation’) or between ES and undifferentiated (MEF) cells (‘activity’) when scoring OCT4 peaks using OCT4 (‘OCT4’) or P300 (‘P300’) distributions. (PDF) [file pcbi.1003342.s017.pdf]

**A Perturbation (HemoChIP)**

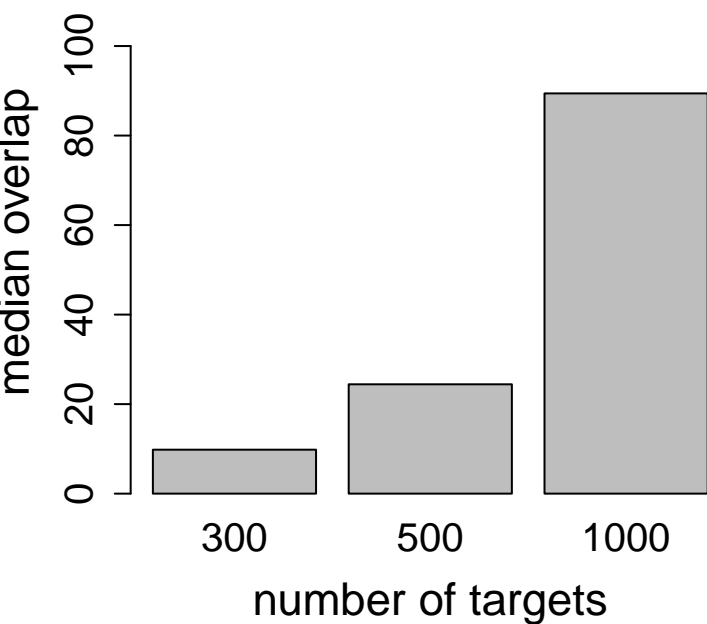

**B Perturbation (ESChIP)**

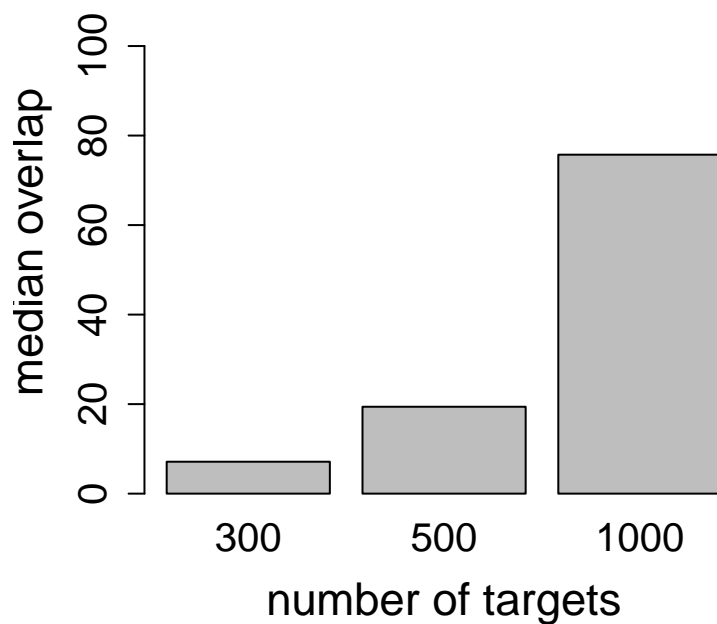

**C Activity (HemoChIP)**

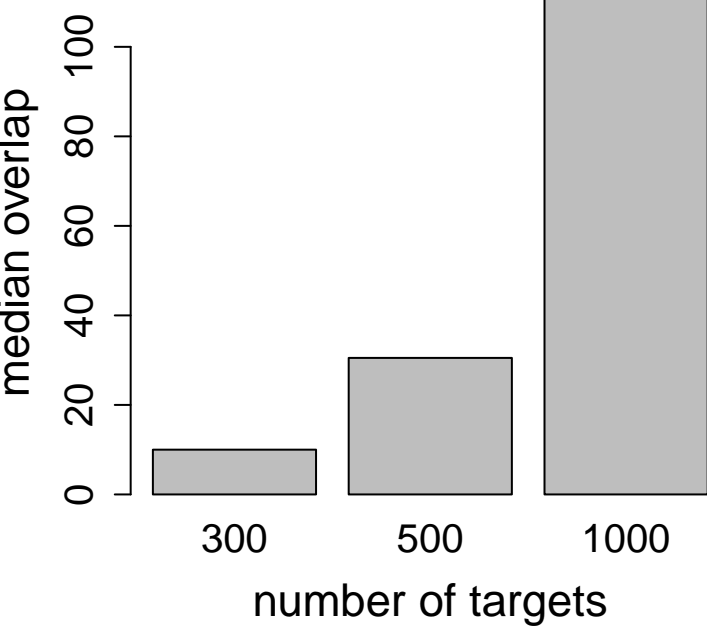

**D Activity (ESChIP)**

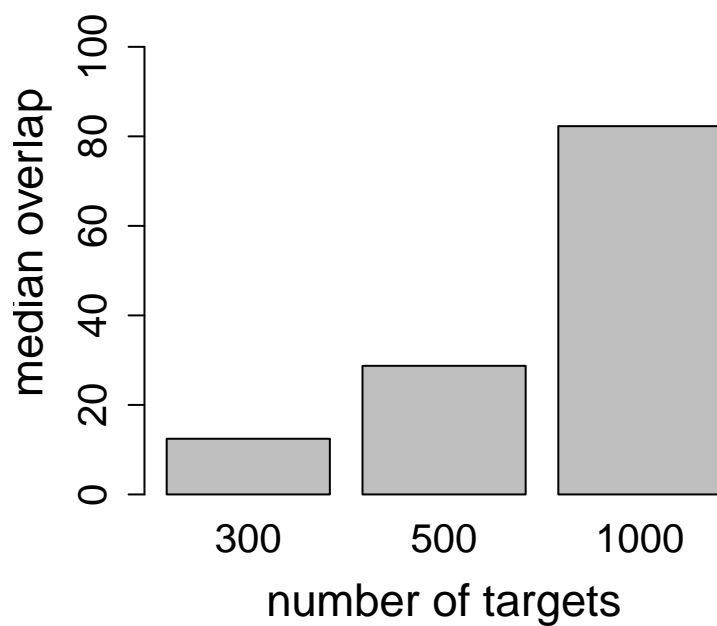

Supplement: Figure S18 — Overlap between targets and differentially expressed genes. Median overlap between top 300, 500 and 1000 targets with the respective number of genes differentially expressed in (A) HemoChIP and (B) ESChIP TF perturbation experiments. Median overlap between top 300, 500 and 1000 targets with the respective number of genes differentially expressed (C) between erythroid and myeloid cells or (D) between undifferentiated (ES) and differentiated (MEF) cells. (PDF) [file pcbi.1003342.s018.pdf]
