# Supplementary material for: Assessing Computational Methods for Transcription Factor Target Gene Identification Based on ChIP-seq Data
Source: PLoS Comput Biol. 2013 Nov 21;9(11):e1003342. doi: 10.1371/journal.pcbi.1003342 (PMC3837635; doi:10.1371/journal.pcbi.1003342)
Supplement: Table S1 — Transcription factors and data included in the study. (PDF) [file pcbi.1003342.s019.pdf]

Table S1. Transcription factors and data included in the study.

Factor	DNA-binding domain [35]	Cell type	PMID of the original ChIP-seq study	Perturbation experiment	Gene Expression Omnibus (GSE) or Array Express accession	Reference for perturbation data
**HemoChIP** [15]						
CEBPA	bZIP	**Mac**	20513432	CEBPA+ and CEBPA- bone marrow LSK cells (*)	GSE23800	[36]
CEBPB	bZIP	**Mac**	20513432	bone marrow-derived macrophages isolated from *Cebpb*-WT and *Cebpb*-KO mice	GSE23821	[37]
CTCF	Zinc finger, C2H2	ES<br>Pre-Adi<br>**Pro-B cell**	20720539<br>20887899<br>20543837	-	-	-
E2A	bHLH	Pre-Pro-B cell<br>**Pro-B cell**	20543837<br>20543837	bone marrow-derived LMPP progenitor cells isolated from *E2a*-WT and *E2a*-KO mice	GSE11982	[38]
EBF	bHLH	**Pro-B cell**	20451411	fetal liver-derived $Ebf^{+/fl}$ and $Ebf^{fl/fl}$ pro-B cells after induction Cre recombinase	E-GEOD-21454	[39]
ERG	ETS-type	**HPC7**	20887958	-	-	-
ETO2	-	Ery-MEL<br>**Ind-Ery-MEL**	20123907<br>20123907	control-siRNA and *Eto2*-siRNA treated induced G1E-ER-GATA-1 cells	E-GEOD-24333	[40]
FLI1	ETS-type	**HPC7**	20887958	-	-	-

Continued on next page

Continued from previous page

FOXO1	Fork head	**Pro-B cell**	20543837	Treg cells isolated from *Foxo1/3*-WT and *Foxo1/3*-KO mice	GSE21678	[41]
GATA1	Zinc finger, GATA	Ery-G1ER<br>**Ery-MEL**<br>Ind-Ery-MEL	19887574<br>20123907<br>20123907	fetal liver-derived progenitors grown into megakaryocytes isolated from *Gata1*-WT and *Gata1*-KO mice	GSE2527	[42]
GATA2	Zinc finger, GATA	eHPC<br>**HPC7**	21186366<br>20887958	-	-	-
GFI1B	Zinc finger, C2H2	**HPC7**	20887958	-	-	-
LDB1	-	**Ery-MEL**<br>eHPC<br>Ind-Ery-MEL	20123907<br>21186366<br>20123907	-	-	-
LMO2	-	**HPC7**	20887958	control-siRNA and *Lmo2*-siRNA treated induced G1E-ER-GATA-1 cells	E-GEOD-24336	[40]
LYL1	bHLH	**HPC7**	20887958	-	-	-
MEIS1	TALE homeodomain	**HPC7**	20887958	-	-	-
MTGR1	-	**Ery-MEL**<br>Ind-Ery-MEL	20123907<br>20123907	-	-	-
OCT2	POU homeodomain	**B cell**	20513432	-	-	-
P300	-	**Mac**	20206554	bone marrow-derived LSK cells isolated from *P300*-WT and *P300*-KO mice	E-TABM-1050	[43]
P65	Rel homology region	**Act.Mac**	21106671	-	-	-
PPARG	Zinc finger, C4	**Mac**	20176806	-	-	-

Continued on next page

Continued from previous page

PU1	ETS-type	**Act.Mac**<br>B cell<br>Mac<br>Mac<br>HPC7	20206554<br>20513432<br>20206554<br>20513432<br>20887958	bone marrow-derived HSC cells isolated from *Pu1*-WT and *Pu1*-KD mice	GSE5654	[44]
RAG2	-	**Thy**	20398922	-	-	-
RUNX1	Runt domain	**HPC7**	20887958	-	-	-
SCL	bHLH	Ery-MEL<br>eHPC<br>**Ind-Ery-MEL**<br>HPC7<br>Ery-FL (RER)<br>Ery-FL (WT)	20123907<br>21186366<br>20123907<br>20887958<br>20887958<br>20887958	fetal liver-derived progenitors grown into megakaryocytes isolated from $Scl^{f}l/fl$ mice, treated and untreated with Cre recombinase	GSE14478	[45]
STAT3	STAT domain	**T cell**	20493732	-	-	-
STAT4	STAT domain	**T cell**	20620946	T cells isolated from *Stat4*-WT and *Stat4*-KO mice	GSE22081	[46]
STAT5A	STAT domain	13h-T cell<br>**2r-T cell**<br>8h-T cell	18820682<br>18820682<br>18820682	-	-	-
STAT5B	STAT domain	13h-T cell<br>**2r-T cell**<br>8h-T cell	18820682<br>18820682<br>18820682	-	-	-
STAT6	STAT domain	**T cell**	20620946	T cells isolated from *Stat6*-WT and *Stat6*-KO mice	GSE22081	[46]
**ESChIP** [12]						
CMYC	bHLH-ZIP	**ES**	-	-	-	-
CTCF	Zinc finger, C2H2	**ES**	-	-	-	-
E2F1	Fork head / winged helix	**ES**	-	T cells isolated from *E2f1*-WT and *E2f1*-KO mice	E-MEXP-1991	[47]
ESRRB	Zinc finger, C4	**ES**	-	control-shRNA and *Esrrb*-shRNA treated ES cells	E-GEOD-13212	[48]

Continued on next page

Continued from previous page

KLF4	Zinc finger, C2H2	**ES**	-	-	-	-
NANOG	Homeodomain	**ES**	-	control-RNAi and *Nanog*-RNAi treated ES cells	GSE4189	[9]
NMYC	bHLH-ZIP	**ES**	-	-	-	-
OCT4	POU homeodomain	**ES**	-	control-RNAi and *Oct4*-RNAi treated ES cells	GSE4189	[9]
P300	-	**ES**	-	-	-	-
SMAD1	SMAD/NF-1 domain	**ES**	-	embryonic lung *Smad1*-WT and *Smad1*-CKO cells	GSE26502	[49]
SOX2	HMG	**ES**	-	control-shRNA and *Sox2*-shRNA neural stem cells	E-MEXP-2743	[50]
STAT3	STAT domain	**ES**	-	-	-	-
SUZ12	-	**ES**	-	ES cells isolated from *Suz12*-WT and *Suz12*-KO mice	GSE31354	[51]
TCFCP2L1	Grainyhead	**ES**	-	-	-	-
ZFX	Zinc finger, C2H2	**ES**	-	*Zfx*-WT and *Zfx*-KO ES cells	GSE7069	[52]

The representative ChIP-seq studies used for target prediction in the majority of the analyses and evaluation tests are marked in bold.
(*) Wild-type LSK cells sorted into CEBPA+ and CEBPA-.

References

35. Stegmaier P, Kel AE, Wingender E (2004) Systematic DNA-binding domain classification of transcription factors. Genome Inform 15: 276–86.

36. Wölfler A, Danen-van Oorschot AA, Haanstra JR, Valkhof M, Bodner C, et al. (2010) Lineage-instructive function of C/EBPα in multipotent hematopoietic cells and early thymic progenitors. Blood 116: 4116–4125.

37. Akagi T, Thoennissen NH, George A, Crooks G, Song JH, et al. (2010) In vivo deficiency of both C/EBPβ and C/EBPϵ results in highly defective myeloid differentiation and lack of cytokine response. PloS One 5: e15419.

38. Dias S, Mansson R, Gurbuxani S, Sigvardsson M, Kee BL (2008) E2A proteins promote development of lymphoid-primed multipotent progenitors. Immunity 29: 217–227.

39. Treiber T, Mandel EM, Pott S, Györy I, Firner S, et al. (2010) Early B cell factor 1 regulates B cell gene networks by activation, repression, and transcription- independent poising of chromatin. Immunity 32: 714–725.

40. Fujiwara T, Lee HY, Sanalkumar R, Bresnick EH (2010) Building multifunctionality into a complex containing master regulators of hematopoiesis. Proc Natl Acad Sci U S A 107: 20429–20434.

41. Ouyang W, Beckett O, Ma Q, Paik JH, DePinho RA, et al. (2010) Foxo proteins cooperatively control the differentiation of Foxp3+ regulatory T cells. Nat Immunol 11: 618–27.

42. Muntean AG, Crispino JD (2005) Differential requirements for the activation domain and FOG-interaction surface of GATA-1 in megakaryocyte gene expression and development. Blood 106: 1223–1231.

43. de Graaf CA, Kauppi M, Baldwin T, Hyland CD, Metcalf D, et al. (2010) Regulation of hematopoietic stem cells by their mature progeny. Proc Natl Acad Sci U S A 107: 21689–21694.

44. Steidl U, Rosenbauer F, Verhaak RGW, Gu X, Ebralidze A, et al. (2006) Essential role of Jun family transcription factors in PU.1 knockdown-induced leukemic stem cells. Nat Genet 38: 1269–1277.

45. Gekas C, Rhodes KE, Gereige LM, Helgadottir H, Ferrari R, et al. (2009) Mef2C is a lineage-restricted target of Scl/Tal1 and regulates megakaryopoiesis and B-cell homeostasis. Blood 113: 3461–71.

46. Wei L, Vahedi G, Sun HW, Watford WT, Takatori H, et al. (2010) Discrete roles of STAT4 and STAT6 transcription factors in tuning epigenetic modifications and transcription during T helper cell differentiation. Immunity 32: 840–851.

47. Infante A, Laresgoiti U, Fernández-Rueda J, Fullaondo A, Galán J, et al. (2008) E2F2 represses cell cycle regulators to maintain quiescence. Cell Cycle 7: 3915–3927.

48. Feng B, Jiang J, Kraus P, Ng JH, Heng JCD, et al. (2009) Reprogramming of fibroblasts into induced pluripotent stem cells with orphan nuclear receptor Esrrb. Nat Cell Biol 11: 197–203.

49. Xu B, Chen C, Chen H, Zheng SG, Bringas J Pablo, et al. (2011) Smad1 and its target gene Wif1 coordinate BMP and Wnt signaling activities to regulate fetal lung development. Development 138: 925–935.

50. Engelen E, Akinci U, Bryne JC, Hou J, Gontan C, et al. (2011) Sox2 cooperates with Chd7 to regulate genes that are mutated in human syndromes. Nat Genet 43: 607–11.

51. Pasini D, Bracken AP, Hansen JB, Capillo M, Helin K (2007) The polycomb group protein Suz12 is required for embryonic stem cell differentiation. Mol Cell Biol 27: 3769–3779.

52. Galan-Caridad JM, Harel S, Arenzana TL, Hou ZE, Doetsch FK, et al. (2007) Zfx controls the self-renewal of embryonic and hematopoietic stem cells. Cell 129: 345–357.
