# Supplementary material for: Assessing Computational Methods for Transcription Factor Target Gene Identification Based on ChIP-seq Data
Source: PLoS Comput Biol. 2013 Nov 21;9(11):e1003342. doi: 10.1371/journal.pcbi.1003342 (PMC3837635; doi:10.1371/journal.pcbi.1003342)
Supplement: Text S1 — Supporting text. (1) TF characterization based on ChIP-seq studies. (2) Importance of using TF-specific peak-to-gene distance distributions. (3) Incorporation of peak height or binding of co-factors does not improve target prediction. (4) Significance and robustness of evaluation methods. (5) Ranking is biased by non-changing genes. (6) Q-value calculation for ClosestGene scores. (PDF) [file pcbi.1003342.s020.pdf]

Assessing computational methods for transcription factor target gene identification based on ChIP-seq data

Weronika Sikora-Wohlfeld[1], Marit Ackermann[1], Eleni G. Christodoulou[1], Kalaimathy Singaravelu[1], Andreas Beyer[1,2,3,*]

1 Biotechnology Center, TU Dresden, Dresden, Germany

2 Center for Regenerative Therapies Dresden, Dresden, Germany

3 University of Cologne, Cologne, Germany

∗ **E-mail: andreas.beyer@uni-koeln.de**

Supporting text

TF characterization based on ChIP-seq studies

In order to systematically characterize the studied transcription factors, we investigated the co-occurrence of TF peaks with proximal promoter regions, P300-bound regions and CTCF-bound regions. First, all peaks identified in ESChIP experiments were converted to 400 bp based on the peak centers, which is then consistent with the HemoChIP data. Promoter regions were defined as 400 bp length regions centered at the TSS of each gene. For each TF, peaks overlapping with these promoter regions were classified as promoter-associated peaks.

Identification of genomic regions bound by diverse TFs within the same cellular system (hematopoietic cells or embryonic stem cells) was done similarly as described in [15]. All peaks across all ChIP-seq studies (separately for HemoChIP and ESChIP) were pooled, the overlapping peaks were merged and a list of non-overlapping regions, bound by at least one TF was generated. The identified regions were subsequently intersected with the original ChIP-seq studies to find which TFs share binding at particular regions. For each TF, peaks that overlapped with the regions bound by P300 or CTCF were defined as P300-associated or CTCF-associated, respectively.

Here, P300 serves as a marker for enhancer regions, which can be far away from promoters, and CTCF binds at insulators, i.e. also often outside of promoter regions. Figure S14 shows a broad diversity among the factors with respect to their binding preferences. The fraction of peaks occurring at promoters (defined as a 400 bp window around the TSSs), varies between 1.5% and 20% for most TFs. The fact that most bindings are not directly at TSSs is consistent with the recently reported observations that TF binding outside promoter regions might also influence gene expression and that different TFs differ in the proportion of proximal and distal binding events [53]. For example, OCT4, SOX2 and NANOG have a tendency to associate with P300, i.e. to bind in enhancer regions [54]. As opposed to that E2F1, ZFX, KLF4, c-MYC and n-MYC show relatively frequent binding at promoters (Figure S14), which is in agreement with previous reports [55, 56] and with the function of MYC regulating the pause-and-release of Pol II at the TSS [57]. Interestingly, our analysis revealed SMAD1 as the TF with the highest fraction of peaks co-occurring with P300 (26%) [58].

The high fraction of RAG2 peaks (39%) binding at promoters draws attention. RAG2 is neither a transcription factor, nor a transcriptional regulator, but a protein involved in the initiation of V(D)J recombination during B and T cell development. Ji et al. [59] explain the co-occurrence of RAG2 binding sites with promoters in the original RAG2 ChIP-seq study: in contrast to RAG1, whose binding is tightly

restricted, RAG2 binds broadly in the genome and its binding sites highly overlap with H3K4me3 marks. Having no direct DNA-binding activity, RAG2 recognizes H3K4me3 marks with its PHD domain. The H3K4me3 modification marks active transcription. Thus, RAG2 binding is a marker of transcriptional activity rather than a transcriptional regulator.

CTCF is a ubiquitously expressed transcription factor involved both in transcriptional repression [60, 61] and activation [62]. CTCF marks insulators [63], i.e. DNA elements preventing the spread of heterochromatin and inhibiting interactions between enhancers and unrelated promoters [64]. Compared to the other ChIP-seq studies we used, CTCF had relatively large numbers of peaks in both, HemoChIP and ESChIP. The fraction of peaks associated with CTCF is relatively low and similar for most TFs, falling in the range of 2% to 17%. A notable exception is FOXO1, whose fraction of peaks co-occurring with CTCF is 77% [15].

Importance of using TF-specific peak-to-gene distance distributions

Transcription factors show different binding characteristics in the proximity of their target genes, i.e. some TFs bind close to the TSSs, whereas the others bind further away [53]. Therefore, a number of target prediction methods have been proposed using TF characteristic binding profiles to score the peaks in the vicinity of the TSSs [8, 12]. Also *ClosestGene* uses such a TF-specific distribution of peak-to-gene distances. In order to investigate whether using TF-specific distributions yields better performance than TF-unspecific distributions we compared the performance (z-score) of the following *ClosestGene* variants differing in the distributions used for the target scoring: (1) using TF-specific distributions and distinguishing upstream and downstream binding; (2) not distinguishing upstream and downstream binding, i.e. only using one TF-specific distribution; (3) using the distribution of another TF; (4) assuming a linear decay of the weights with distance from the TSS (Figure S15). Whereas the test based on the perturbation expression data does not show significant differences between the performance of different variants (Figure S15 A, B), the test based on the expression data from different physiological conditions shows differences between the variants (Figure S15 C, D). This could be explained by the fact that the activity-based test is based on a larger number of TFs (all considered TFs), whereas the perturbation-based test includes only those TFs, for which corresponding expression data were available. The analysis shows that (1) distinguishing upstream and downstream binding yields a small improvement of the scoring and (2) simply assuming a linear decay of weights yields worse results compared to using the

actual binding distribution.

Surprisingly, we did not observe a significant difference between *ClosestGene* using a TF-specific distribution and *ClosestGene (unspecific)* where a distribution specific for another TF was used. This observation might be explained by the fact that the above analysis might be dominated by the majority of TFs in this study having similar peak-to-gene distance distribution. However, in cases where the distributions are very different using the correct (TF-specific) distribution might be important. In order to test that hypothesis we specifically analysed the distribution of OCT4 and P300, which are examples of two very different distributions (Figure S16). In this particular case we observed a decrease in performance when using P300's distribution to call OCT4 targets (Figure S17).

This example shows that using TF-specific distributions is advantageous. Further, using the peak-to-gene distance distributions eliminates the necessity of making any arbitrary assumptions about the peak influence of gene expression (e.g. linear or exponential decay) or choosing any arbitrary parameters such as window size. Finally, distinguishing upstream and downstream peaks, which is often neglected by simple target prediction approaches, gives better results than using a symmetric distribution (Figure S15).

Incorporation of peak height or binding of co-factors does not improve target prediction

In the analysis presented in the main text, *ClosestGene* peak scoring was just based on the distance of peaks from genes. Additionally, we tested if considering co-binding of other TFs or peak intensities would improve the target prediction. Identification of genomic regions bound by diverse TFs was done similarly as described in [15]. All peaks across all TFs (together for HemoChIP and ESChIP) were pooled, the overlapping peaks were merged and a list of non-overlapping regions, bound by at least one TF was generated. The identified regions were subsequently intersected with the original peaks to find which TFs share binding at particular regions. An occupancy score was computed by counting the number of TFs binding at each site. Next, each site was scored based on the fraction of sites with the same or higher occupancy. Thus, this score reflects the probability of obtaining such site by randomly drawing a site from all sites. Finally, peaks were scored using the site-specific score either alone or in combination with the distance score. To quantify the peak intensity, for each TF peaks were scored using the fraction of peaks with the same or higher intensity value (specifically for each TF). Thus, this fraction reflects

the probability of obtaining this intensity score by chance when randomly drawing a peak from all peaks of that ChIP-seq study. These scores were then integrated with distance-based and/or co-binding-based scores.

We observed that peaks binding at sites that are also co-bound by other factors tend to be closer to TSSs than isolated peaks (Figure S10). Note that Chen *et al.* [12] reported a depletion of co-binding events at promoters. Here, however, we are looking at larger regions of up to 1 Mb around the TSSs. Likewise, we observed that peaks with higher intensities tend to be closer to TSSs for ESChIP TFs (Figure S11 B). In the case of HemoChIP this effect could not be observed. The difference between HemoChIP and ESChIP can possibly be explained by higher quality of measurements and processing of ESChIP data.

Based on these observations we reasoned that genomic regions bound by multiple TFs and/or with stronger intensities are more likely to have a regulatory function than sites observed in just one experiment or with low peak intensity. Our scoring computes the probability that a randomly selected binding site has at least the observed number of co-binders. This probability can be incorporated in the *ClosestGene* score. Similarly to co-binding of other TFs, we incorporated peak intensity: for ChIP-seq datasets with available peak intensity information we computed the probability of each individual peak having the observed or higher intensity. Figure S12 shows the results for scoring peaks based on distance, co-binding, peak intensity, and various combinations of these criteria. Scoring peaks based on co-binding or intensity alone yields predictions that are worse than using the distance criterion. Although combining the features gives better results it never significantly exceeds the performance of the distance based prediction. In the remainder of this study we therefore did not use peak intensity or co-binding information.

Significance and robustness of evaluation methods

In order to prove that the overlap observed between top targets and top ranking genes in the perturbation and activity analysis is better than random, we randomized the ranking of the genes according to the expression fold change and calculated the resulting overlap. We repeated this procedure 1000 times and used the obtained distributions to calculate the z-scores of the overlaps observed in the real data. Those z-scores are skewed towards positive values (Figure S4), whereas a distribution centered at zero is expected if the overlaps were random.

In order to check if the results are robust and do not depend on the particular subset of top 500

targets, we repeated the analysis using the top 300 and 1000 targets. Figures S5 and S6 confirm the conclusions drawn from the evaluation of TF target prediction methods with expression data: The highest concordance between the target predictions and genes differentially expressed in perturbation experiments or in different physiological conditions is yielded by the methods taking account number of peaks assigned to a gene and their relative distance from the TSS. *ClosestGene*, *Linear*, *Ouyang* and *Cheng* methods perform consistently well across different sets of target genes. Contrary, *Binary* and *Chen* methods perform considerably worse.

In the main text we chose to present the results of the evaluation tests using the top 500 targets. We reason that this threshold allows to obtain robust results maintaining specificity. While 1000 might seem to be too large number of targets for many TFs, choosing less than 300 genes as targets yields overlaps that are too small to draw any reliable conclusions (Figure S18).

Similarly to the evaluation tests based on the expression data, we checked the robustness of the consistency analysis by repeating the test using different sets of targets (300 and 1000). Figure S9 confirms that the conclusions from this evaluation test do not depend on the number of top targets selected.

In order to check the robustness of the evaluation test based of the functional homogeneity of the targets, we repeated the assessment using three different sets of targets (Figure S7) and two different p-value thresholds for significant GO terms (Figure S8). The analysis revealed that *ClosestGene* consistently finds the highest number of specific GO terms.

Finally, we checked the targets regions gene density for different sets of targets. Again, the conclusions were independent from the selected set of targets (Figure S13).

Ranking is biased by non-changing genes

As an alternative to the analysis of the overlap between sets of top genes, we tested assessing the consistency by computing the sum of products of each gene's ranks in the two ranked lists [19]. We also tested the Spearman rank correlation as another measure scoring the whole ranking and it performed very similar to the sum of rank products (not shown). For each TF, for which perturbation expression data were available, all genes were ranked according to the TF-gene association score and according to the expression fold change. Subsequently, we computed sums of rank products for each ChIP-seq - expression data pair and compared the resulting sums to randomized rankings. We noticed that the scores were on

average better than random. However, we also noticed that pairing ChIP-seq studies with non-matching expression data resulted in scores better than random. This was even true for ChIP-seq data from ES-ChIP paired with HemoChIP expression data and vice versa. We hypothesized that this result may be an artifact due to genes that do not change their expression under any of the tested conditions. Especially house-keeping genes that are not regulated by any TF will get low target scores for any ChIP-seq study irrespective of the TF tested.

In order to investigate this problem more in detail we performed the following analysis. For each TF for which perturbation expression data were available, the overlap between the top 500 genes ranked according to the TF-gene association score and top 500 genes ranked according to the expression fold change as well as the sum of the rank products were calculated. The same analysis was repeated replacing the perturbation dataset specific for a given TF with all the other available perturbation datasets specific for other TFs. Subsequently, a one-sample one-sided t-test was performed between the result (overlap or sum of the rank products) obtained for the matching ChIP-seq dataset - perturbation dataset pair and the results obtained for all ChIP-seq dataset - perturbation datasets specific for different, but belonging to the same category (HemoChIP or EsChIP) TFs.

Similarly, a one-sample one-sided t-test was performed between the result obtained for the matching ChIP-seq dataset - perturbation dataset pair and the results obtained for all ChIP-seq dataset - perturbation datasets specific for TFs belonging to the other category pairs. The procedure was repeated for all compared TF-target prediction methods and the $-log_{10}$(p-value) across all tests were pooled and summarized with boxplots. This analysis is similar to the one shown in Figure 4 of the main text.

To perform the above analysis the perturbation expression datasets were processed in three ways: (1) all genes which were scored by TF-target prediction methods and profiled in the perturbation experiments were included in the analysis, (2) only the top 5000 most variable genes were included in the analysis, (3) only the top 2000 most variable genes were included in the analysis. In order to select variable genes the fold changes across all perturbation experiments were first quantile normalized. Next, the genes were sorted according to the maximal fold change observed in any experiment. Finally, the 5000 or 2000 most variable genes were selected. This filtering selects genes that are responding under at least one condition, which are thus under regulatory control and potential TF targets.

Figure S3 shows that this filtering significantly increases the specificity of the predictions when using the rank products for scoring, especially when comparing ESChIP versus HemoChIP and vice versa

('other category'). The consistency analysis based on the top 500 genes is different in two ways: first, it achieves much higher specificity to begin with (compare 'all' in Panel B with 'all' in Panel D). Second, filtering for variable genes does not further improve the specificity. Thus, measuring the overlap of the top 500 ranking genes is less affected by unspecific correlation between ChIP-seq and expression data.

Q-value calculation for *ClosestGene* scores

The original *ClosestGene* TF-target scores depend on the overall number of peaks in the ChIP-seq study: Studies with more peaks will generally yield higher scores, because on average more peaks are assigned to the genes. We addressed this problem through permutation testing, which we used to determine false discovery rates (FDR) and q-values [65]. In order to estimate the distribution of the *ClosestGene* scores under the null hypothesis that none of the genes is a target, we shuffled the peak-to-gene assignment. Based on this random assignment the *ClosestGene* score was calculated for each gene and the procedure was repeated 500 times. The scores calculated under the null hypothesis were pooled and the resulting distribution was used to transform the original scores into FDR corrected q-values. Calculation of the q-values is implemented in the `TFTargetCaller` package (available at www.cellularnetworks.org).

References

53. Massie CE, Mills IG (2008) ChIPping away at gene regulation. EMBO Rep 9: 337–343.
54. Goke J, Jung M, Behrens S, Chavez L, O'Keeffe S, et al. (2011) Combinatorial binding in human and mouse embryonic stem cells identifies conserved enhancers active in early embryonic development. PLoS Comput Biol 7: e1002304.
55. Xu X, Bieda M, Jin VX, Rabinovich A, Oberley MJ, et al. (2007) A comprehensive ChIP-chip analysis of E2F1, E2F4, and E2F6 in normal and tumor cells reveals interchangeable roles of E2F family members. Genome Res 17: 1550–1561.
56. Zeller KI, Zhao X, Lee CW, Chiu KP, Yao F, et al. (2006) Global mapping of c-Myc binding sites and target gene networks in human B cells. Proc Natl Acad Sci U S A 103: 17834–9.
57. Rahl PB, Lin CY, Seila AC, Flynn RA, McCuine S, et al. (2010) c-Myc regulates transcriptional pause release. Cell 141: 432–45.
58. Pearson KL, Hunter T, Janknecht R (1999) Activation of Smad1-mediated transcription by p300/CBP. Biochim Biophys Acta 1489: 354–64.
59. Ji Y, Resch W, Corbett E, Yamane A, Casellas R, et al. (2010) The in vivo pattern of binding of RAG1 and RAG2 to antigen receptor loci. Cell 141: 419–431.
60. Lobanenkov VV, Nicolas RH, Adler VV, Paterson H, Klenova EM, et al. (1990) A novel sequence-specific DNA binding protein which interacts with three regularly spaced direct repeats of the CCCTC-motif in the 5'-flanking sequence of the chicken c-myc gene. Oncogene 5: 1743–53.

61. Burcin M, Arnold R, Lutz M, Kaiser B, Runge D, et al. (1997) Negative protein 1, which is required for function of the chicken lysozyme gene silencer in conjunction with hormone receptors, is identical to the multivalent zinc finger repressor CTCF. Mol Cell Biol 17: 1281–8.

62. Vostrov AA, Quitschke WW (1997) The zinc finger protein CTCF binds to the APBbeta domain of the amyloid beta-protein precursor promoter. evidence for a role in transcriptional activation. J Biol Chem 272: 33353–9.

63. Bell AC, West AG, Felsenfeld G (1999) The protein CTCF is required for the enhancer blocking activity of vertebrate insulators. Cell 98: 387–396.

64. Kim TH, Abdullaev ZK, Smith AD, Ching KA, Loukinov DI, et al. (2007) Analysis of the vertebrate insulator protein CTCF-binding sites in the human genome. Cell 128: 1231–1245.

65. Benjamini Y, Hochberg Y (1995) Controlling the false discovery rate: A practical and powerful approach to multiple testing. J R Stat Soc Series B Stat Methodol 57: 289–300.
